# Antibacterial Chemodynamic Therapy: Materials and Strategies

**DOI:** 10.34133/bmef.0021

**Published:** 2023-07-17

**Authors:** Chenyang Jia, Fu-Gen Wu

**Affiliations:** State Key Laboratory of Digital Medical Engineering, Jiangsu Key Laboratory for Biomaterials and Devices, School of Biological Science and Medical Engineering, Southeast University, Nanjing 210096, China.

## Abstract

The wide and frequent use of antibiotics in the treatment of bacterial infection can cause the occurrence of multidrug-resistant bacteria, which becomes a serious health threat. Therefore, it is necessary to develop antibiotic-independent treatment modalities. Chemodynamic therapy (CDT) is defined as the approach employing Fenton and/or Fenton-like reactions for generating hydroxyl radical (•OH) that can kill target cells. Recently, CDT has been successfully employed for antibacterial applications. Apart from the common Fe-mediated CDT strategy, antibacterial CDT strategies mediated by other metal elements such as copper, manganese, cobalt, molybdenum, platinum, tungsten, nickel, silver, ruthenium, and zinc have also been proposed. Furthermore, different types of materials like nanomaterials and hydrogels can be adopted for constructing CDT-involved antibacterial platforms. Besides, CDT can introduce some toxic metal elements and then achieve synergistic antibacterial effects together with reactive oxygen species. Finally, CDT can be combined with other therapies such as starvation therapy, phototherapy, and sonodynamic therapy for achieving improved antibacterial performance. This review first summarizes the advancements in antibacterial CDT and then discusses the present limitations and future research directions in this field, hoping to promote the development of more effective materials and strategies for achieving potentiated CDT.

## Introduction

Bacterial infection, which results from harmful bacteria invading the host, is a common cause of mortality and morbidity and an urgent health problem in the world [1–4]. Moreover, bacteria attaching to surfaces can aggregate in a hydrated polymeric matrix synthesized by the bacteria themselves to construct biofilms. Establishment of these sessile communities and their intrinsic resistance to antimicrobial agents are also the causes of many chronic and persistent bacterial infections [5]. To treat a bacterial infection, using antibiotics such as penicillin is the first step [6]. However, the frequency of resistance is elevated in many types of bacteria, which is caused by increasing antimicrobial uses. The treatment of multidrug-resistant (MDR) strains sometimes needs the use of 6 to 7 different drugs [7]. MDR bacteria have been recently listed as the fifth threat to human health and are ranked third on the report of global public health threats [8]. To solve the problems caused by the current antibiotic treatment for bacterial infections, researchers have made many efforts to develop novel anti-infection strategies. For example, a variety of nanomaterials that can cause disruption of bacterial cell wall and membrane have been developed for killing bacteria [9]. There are also some antibacterial treatments that are achieved by generating reactive oxygen species (ROS). ROS have the capacity to inactivate a variety of pathogens [10]. Common ROS include singlet oxygen (^1^O_2_), hypochlorous acid (HClO), hydrogen peroxide (H_2_O_2_), superoxide anion (O_2_^•−^), and hydroxyl radical (•OH) [11]. In addition, some types of nanoparticles (NPs) have been engineered to interfere with the cellular homoeostasis and intracellular signaling pathways of bacterial cells, ultimately leading to the cell death [9].

Chemodynamic therapy (CDT), which was proposed as a novel therapeutic strategy in 2016, is defined as the therapy employing Fenton or Fenton-like reaction for producing hydroxyl radical (•OH) in the lesion area [12–15]. Fenton reaction is the reaction between iron (Fe) and hydrogen peroxide (H_2_O_2_), which can engender toxic •OH and a higher oxidation state of Fe [16]. Recently, CDT has received more and more attention due to its unique advantages such as low side effects and simple/no requirement of therapeutic equipment. Therefore, several reviews focusing on the application of CDT in tumor treatment have been published [17–19].

**Fig. 1.**
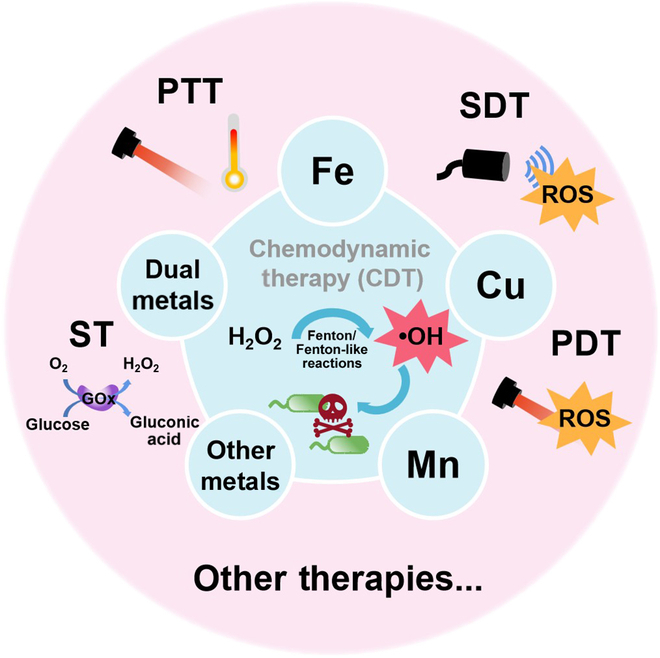
Fenton/Fenton-like reactions in CDT and use of CDT and other therapies for antibacterial treatments.

Since •OH, one of the products of Fenton/Fenton-like reactions, is also a type of ROS, many researchers have applied CDT to the ROS-based antibacterial treatments (Fig. 1). In a CDT-based treatment, Fe-containing materials release ferrous ions (Fe^2+^) in the infection area and evoke Fenton reaction to generate •OH, which can finally lead to bacterial death. Besides Fe, other metal elements can also induce Fenton-like reactions and therefore be employed in CDT processes. These metal elements include cobalt (Co), manganese (Mn), copper (Cu), etc [13]. CDT used in antibacterial treatment has the following characteristics: (a) Toxicities associated with some metals used in CDT may be caused by ROS-mediated cellular damage, and diverse metal-catalyzed oxidation reactions can be the base for the damage of protein, specific types of DNA, or cell membrane [20]. (b) Some metals that are introduced during the process of CDT can also be toxic to bacteria through other mechanisms, thus realizing synergistic antibacterial effects. These mechanisms include protein dysfunction, impaired membrane function, interference with nutrient assimilation, etc [20]. Besides, more antibacterial strategies have been developed through combining CDT with other therapies for achieving better treatment effects.

In this review, we will introduce the progress in CDT-based antibacterial therapies in recent years (some of which are summarized in Table 1) and propose the current challenges as well as limitations in this field.

**Table 1.** Different types of materials for CDT and CDT-involved antibacterial treatments.

Types of material	Metal elements	Types of therapy	CDT agents	Reference
Nanomaterial	Fe	CDT	FePS_3_	[24]
			Ferrocene	[29]
			Amorphous iron	[31]
		CDT and PTT	Fe_3_O_4_	[41]
			Fe-N-C SAzyme	[44]
		CDT and ST	Single-atom Fe	[58]
		CDT and antibiotic therapy	Fe^3+^-doped MOFs	[74]
		CDT, antibacterial peptide treatment, and antibiotic therapy	MIL-101	[78]
	Cu	CDT	CuO_2_	[87]
		CDT and PTT	Cu single-atom site	[106]
			Cu-BIF	[107]
			Cu_2_O	[108]
		CDT and ST	Cu-doped ZIF-8	[115]
	Mn	CDT and chemical therapy	MnO_2_	[129]
		CDT and PTT	Mn single-atom catalyst	[130]
	Co	CDT and PDT	ZIF-67	[139]
	Mo	CDT and PTT	Mo/W POM	[141]
	W	CDT, PTT, and antibiotic therapy	WS_2_ quantum dots	[148]
	Ni	CDT and PTT	ND	[150]
	Fe and Cu	CDT	CuFe_5_O_8_	[160]
	Fe and Cu	CDT, PTT, and PDT	CuFe_2_O_4_	[161]
	Pt and Au	CDT and PTT	Gold–platinum nanodot	[166]
	Cu and Pt	CDT, PTT, and ST	Cu_2_O/Pt nanozyme	[167]
Gel	Cu	CDT	Cu^2+^-loaded ionic gel	[89]
Hydrogel	Fe	CDT	Ferrocene	[30]
			Hb	[33]
		CDT and PTT	Fe^3+^–EDTA complex	[42]
			Fe-doped bioactive glass NPs	[43]
		CDT, PTT, and ST	MnFe_2_O_4_	[79]
	Cu	CDT and PTT	Cu^2+^-containing complex	[109]

## Applications of CDT in Antibacterial Treatments

### Iron-mediated CDT

As mentioned above, Fe^2+^ can react with H_2_O_2_ and then generate •OH for bacterial inactivation. To date, there have been extensive researches on Fe-containing antibacterial materials. This part will introduce various bacterial treatment approaches based on Fe-involved CDT, some of which are combined with other therapies for achieving better antibacterial effects.

#### Single CDT

**Fig. 2.**
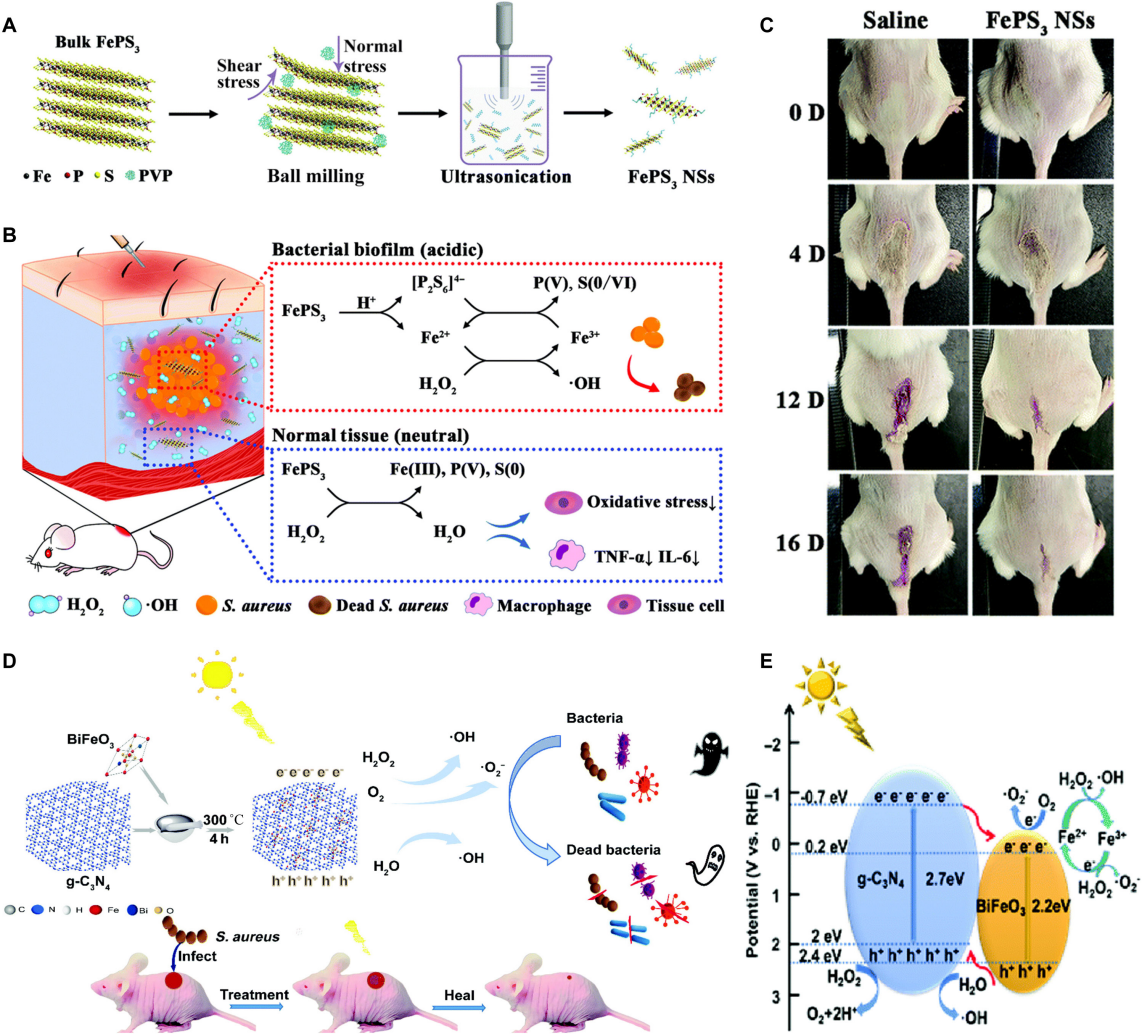
(A) Preparation procedure of FePS_3_ NSs through the ball milling and ultrasonication method. (B) Working mechanisms of FePS_3_ NSs for pH-responsive CDT in biofilm-infected sites and their ROS-depleting property in normal tissues. (C) Photographs of the infected wounds after different treatments. (A to C): Adapted from [24] with permission. Copyright 2021, The Royal Society of Chemistry. (D) Fabrication process and action mechanisms of BFO/CN in antibacterial treatment and wound healing. (E) Diagram showing the photo-Fenton reaction mechanism of the BFO/CN composite. (D and E): Adapted from [32] with permission. Copyright 2022, The Royal Society of Chemistry.

Different from the planktonic bacteria causing acute infections [21], biofilms typically cause chronic infections, which are mainly due to the inflammatory responses [22]. The generation of ROS is considered to be the key event in the progression of various inflammatory disorders. Persistent and uncontrolled oxidative stress with overproduction of ROS will cause apoptosis and tissue injury [23]. Therefore, it is important to develop a treatment modality with both antibiofilm and anti-inflammatory effects. Fortunately, some iron-containing materials can trigger Fenton reaction and then produce toxic •OH to damage biofilms [24,25]. For example, Li et al. [24] prepared FePS_3_ nanosheets (NSs) from bulk FePS_3_ via ball milling and subsequent ultrasonic exfoliation, and the NSs exhibited Fenton activity for antibiofilm use and ROS scavenging capacity for anti-inflammation purpose (Fig. 2A and B). In this system, FePS_3_ NSs showed acid-responsive dissociation, therefore releasing Fe^2+^ and [P_2_S_6_]^4−^ in biofilms with acidic microenvironments [26–28], while under neutral conditions, FePS_3_ was relatively stable. To realize CDT, the released Fe^2+^ then converted H_2_O_2_ to •OH via Fenton reaction for killing the bacteria in biofilms. In addition, the reductive [P_2_S_6_]^4−^ reduced the as-formed ferric ions (Fe^3+^) to Fe^2+^, which could accelerate Fe redox cycling and elevate the activity of Fenton reaction. In the normal tissues with a neutral pH, the FePS_3_ NSs exhibited antioxidative ability and could deplete •OH or H_2_O_2_ by the [P_2_S_6_]^4−^-mediated redox reaction, which exerted an anti-inflammation effect. In vivo experiments indicated that FePS_3_ NSs could effectively promote the recovery of biofilm-infected tissues (Fig. 2C). This work develops a type of pH-responsive CDT nanoagent for realizing both antibiofilm and anti-inflammatory therapies.

Besides, other types of iron-containing materials have also been reported as CDT agents for bacterial treatment [29–34]. For example, Park et al. [29] designed a kind of antibacterial agents composed of a H_2_O_2_-generating polymer (poly[(3-phenylprop-2-ene-1,1-diyl)bis(oxy)bis(ethane-2,1-diyl)diacrylate]-*co*-4,4′(tri-methylene dipiperidine)-*co*-poly(ethylene glycol), abbreviated as PCAE) and iron-containing ferrocene (Fc). Amphiphilic PCAE was fabricated to incorporate H_2_O_2_-producing cinnamaldehyde (CA) moieties and self-assemble to construct thermodynamically stable micelles that encapsulated Fc in their hydrophobic core. When encountering the bacteria-infected site with a low pH (which is caused by the rapid proliferation and high metabolic rate of bacteria [35]), PCAE micelles could release CA and Fc. Then, the CA induced the production of H_2_O_2_ in bacteria and Fe in Fc rapidly converted H_2_O_2_ into highly toxic •OH for CDT. Overall, this work successfully develops a polymeric micelle with Fenton reaction activity and demonstrates its application potential for antibacterial treatment. In addition, Peng and coworkers [30] designed an antibacterial hydrogel dressing (termed Fc-PAAM) with strong adhesion property. Fc-PAAM was composed of a polyacrylamide hydrogel framework as well as Fc-linked polyacrylic acid. When exposed to exogenous H_2_O_2_ with low concentrations, the Fc-PAAM hydrogel showed an excellent peroxidase (POD)-like activity and produced massive •OH for CDT, causing the destruction of bacterial cell membrane and then the death of the bacteria. In vivo experiments further exhibited that the hydrogel could effectively repair bacterially infected wounds, facilitate the generation of epithelial cells in the wound area, and accelerate healing. Further, to elevate CDT's efficacy, 2 external stimuli, the alternating magnetic field (AMF) [31] and the light [32], have been used in bacterial treatments. Gao et al. [31] constructed a kind of amorphous iron NPs (AIronNPs) via reducing Fe^3+^ in poly(ethylene glycol)-*block*-poly(propylene glycol)-*block*-poly(ethylene glycol) (Pluronic F127) bubbles containing poly(vinylpyrrolidone) (PVP). These PVP-functionalized AIronNPs were employed as the antibacterial agents. Moreover, under the AMF, the liberation of Fe^2+^ from AIronNPs was accelerated. Therefore, the catalytic activity of AIronNPs was enhanced, which could generate extra •OH for improved CDT. In the in vivo experiments, these NPs promoted wound disinfection and elevated healing efficiency in the presence of an AMF. The authors proved that the AMF could enhance the effect of the antibacterial CDT mediated by AIronNPs. Additionally, Yin et al. [32] employed a mushroom-shaped BiFeO_3_/g-C_3_N_4_ (BFO/CN) composite for antibacterial use and promotion of wound healing (Fig. 2D). This study exhibited that the BFO/CN composite could produce O_2_^•−^ and •OH under 420-nm light illumination, which were used to inhibit the growth of *Staphylococcus aureus* (*S*. *aureus*) and *Escherichia coli* (*E*. *coli*). Furthermore, H_2_O_2_ was added as a coreagent to further potentiate the photocatalytic capacity of BFO/CN. The process of charge transfer and the further photo-Fenton reaction mechanism were described as follows (Fig. 2E): (a) Under visible light illumination, the photo-induced electrons produced on the conduction band (CB) of g-C_3_N_4_ were transferred to the CB of BiFeO_3_. While the h^+^ generated on the valence band (VB) of the BiFeO_3_ could be transferred to the VB of g-C_3_N_4_. The e^−^ accumulated on the CB of BiFeO_3_ showed good reducibility, which could effectively reduce the oxygen molecules and produce a variety of antibacterial substances, including H_2_O_2_, O_2_^•−^, •OH, etc. (b) Fe^3+^ on the surface of BFO/CN was irradiated with visible light to produce Fe^2+^, which reacted with H_2_O_2_ to produce •OH for CDT. ROS such as O_2_^•−^ and •OH generated in the photocatalysis process and photo-Fenton reaction could effectively damage the bacterial cell wall and lead to cell lysis and death. The results of in vivo experiments proved that BFO/CN could effectively enhance anti-infection and wound healing efficiencies. This work highlights the synergistic effect of photocatalysis and photo-Fenton reaction in antibacterial therapy and wound healing promotion.

#### Combination of CDT and PTT

**Fig. 3.**
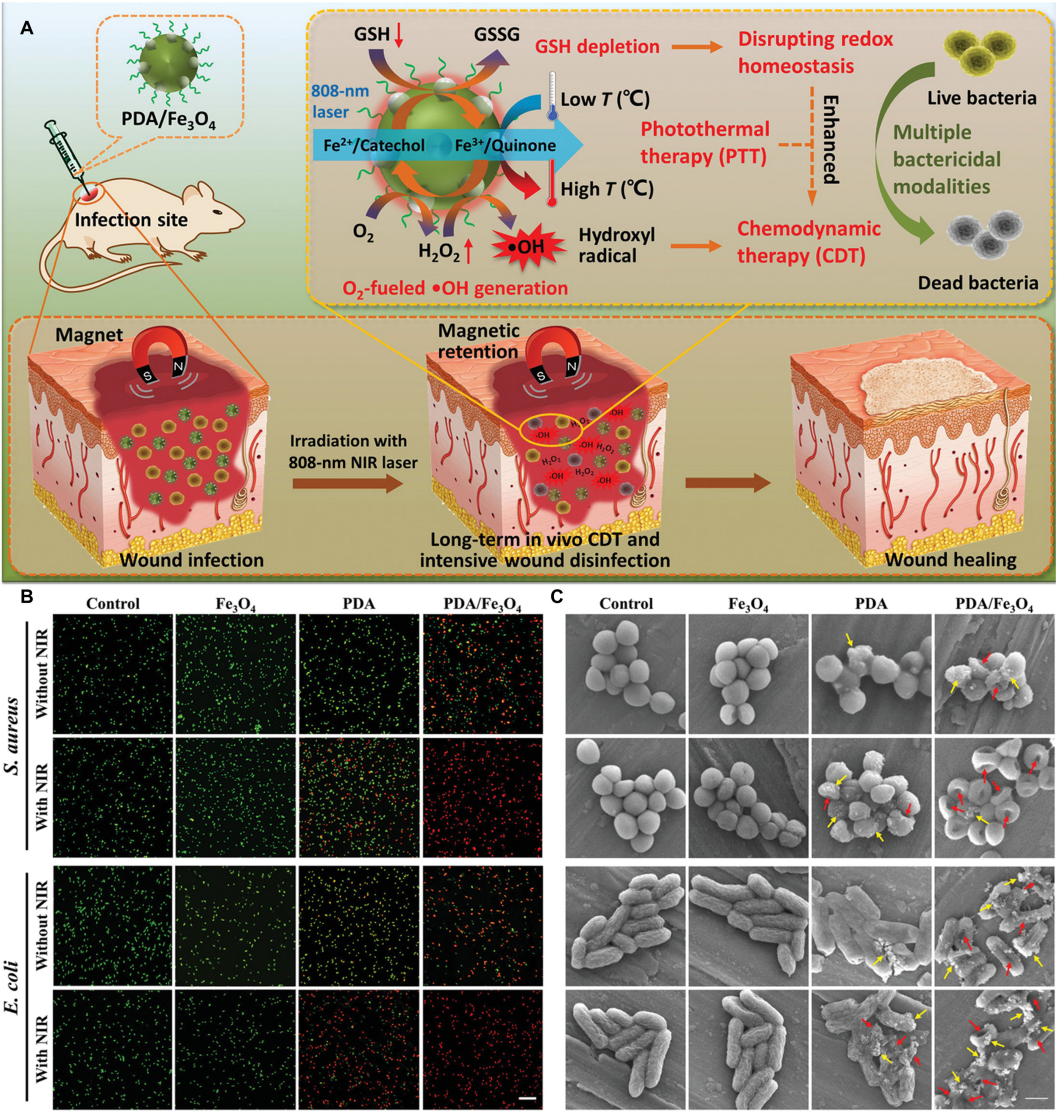
(A) Working mechanism of the PDA/Fe_3_O_4_ hybrid nanozyme for magnetically localized long-term CDT. (B) Confocal fluorescence images of *S*. *aureus* and *E*. *coli* stained with calcein-acetoxymethyl ester/propidium iodide (calcein-AM/PI) after different treatments. (C) Scanning electron microscopy images of *S*. *aureus* and *E*. *coli* treated with various materials. The yellow arrows show the presence of PDA and PDA/Fe_3_O_4_ on the bacterial surface. (A to C): Adapted from [41] with permission. Copyright 2022, Wiley-VCH.

Photothermal therapy (PTT), which can convert near-infrared (NIR) light energy into heat energy, is an excellent alternative to conventional antibacterial strategies because of its minimal invasiveness, negligible toxicity to normal tissues, and high spatiotemporal precision [36,37]. The heat generated during PTT can destroy bacteria via protein denaturation and cell surface disruption, thus resulting in bacterial cell death [38,39]. Besides, the heat is capable of accelerating Fenton reaction and can lead to enhanced antibacterial efficacy. As a result, the synergy of PTT and CDT stands for an important research direction in bacterial treatment. Researchers have employed different types of materials (nanomaterials and hydrogels) to realize this combination [40–55]. For instance, Liu et al. [40] developed a carbon–iron oxide nanohybrid with a rough surface (rough C–Fe_3_O_4_, termed RCF) for realizing improved bacterial adhesion and an NIR-II light-responsive antibacterial treatment. Rough carbon nanoshells showed outstanding abilities of NIR-II light absorption and photothermal conversion and were employed as photothermal agents. Fe_3_O_4_ NPs, as a highly effective POD mimic, were assembled onto the carbon nanoshells and used as nanozymes for CDT. RCF could catalyze H_2_O_2_ to generate toxic •OH. Because of the POD-like catalytic activity of Fe_3_O_4_ NPs and the photothermal property of carbon nanoshells, synergistic CDT/PTT could be realized under mild NIR-II light irradiation. Moreover, RCF with a rough surface showed excellent bacterial adhesion, which benefited both PTT and CDT through the effective interaction between bacteria and RCF. The in vivo experimental results proved the feasibility of adopting RCF to treat methicillin-resistant *S. aureus* (MRSA)-infected rats. In another study, Xiao and coworkers [41] also employed Fe_3_O_4_ to construct a PTT/CDT antibacterial system (Fig. 3A). It is known that glutathione (GSH), as a reductant, can directly scavenge ROS, therefore reducing the efficiency of an ROS-based therapy [56]. Therefore, GSH can also severely reduce the efficacy of CDT. In the work of Xiao et al., the authors prepared a multifunctional hybrid nanozyme (termed PDA/Fe_3_O_4_) consisted of polydopamine (PDA) NPs that were surface-decorated with ultrasmall Fe_3_O_4_ NPs. This nanosystem was designed for enhancing the CDT efficacy through simultaneous GSH consumption and H_2_O_2_-self-sufficient •OH production in infected regions and realizing the localized long-term antibacterial therapy through simple magnetic retention. In particular, the Fe^3+^/Fe^2+^ redox couples in PDA/Fe_3_O_4_ showed Fenton catalytic activity to decompose H_2_O_2_ into •OH for CDT. These Fe^3+^/Fe^2+^ couples also exhibited GSH-depleting capacity to strengthen the CDT-caused oxidative damage. The catechol moieties of PDA could provide electrons to O_2_ to afford H_2_O_2_ that was immediately decomposed by the decorated Fe_3_O_4_ to generate more •OH. The produced quinone groups in PDA were then reduced by GSH, leading to the GSH consumption. Furthermore, because of the superb photothermal conversion efficacy of PDA/Fe_3_O_4_, the heat generated from PTT process could promote the above reactions for enhancing CDT. Additionally, the PDA/Fe_3_O_4_ nanozymes could be restricted in the infection regions with the help of an external magnet to realize long-term in vivo CDT. Both in vitro (Fig. 3B and C) and in vivo studies showed that the PDA/Fe_3_O_4_ possessed satisfactory antibacterial effect due to its capacity to realize combined PTT and CDT. 

**Fig. 4.**
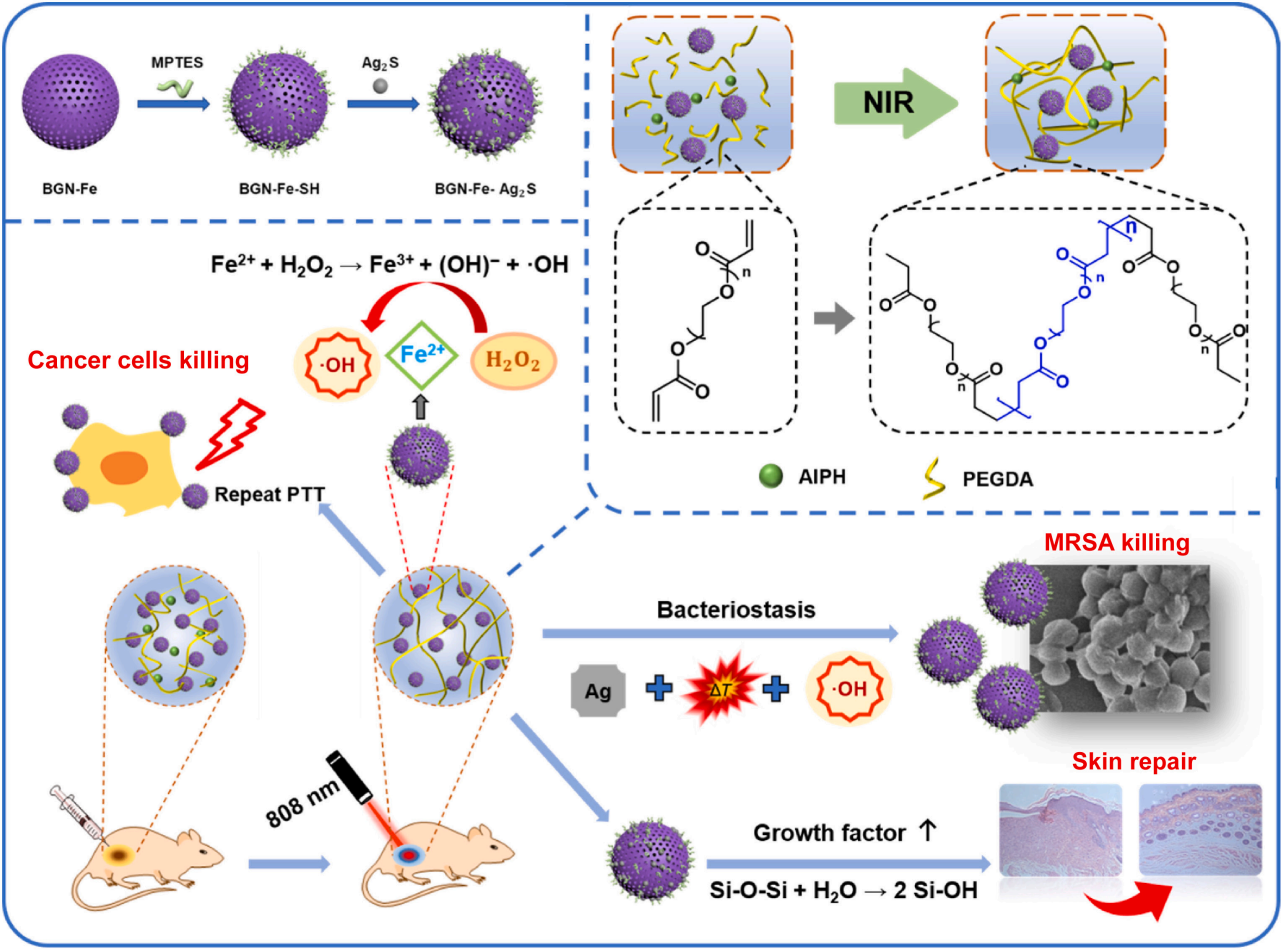
Illustration showing the preparation processes of BGN-Fe-Ag_2_S and PBFA hydrogel and the working mechanism of PBFA hydrogel in synergistic photothermal-chemodynamic cancer therapy and anti-infection treatment. Adapted from [43] with permission. Copyright 2022, Elsevier.

Besides nanomaterials, different types of iron-containing hydrogels can also be designed to achieve antibacterial CDT. For instance, Lin et al. [42] constructed a bacteria-triggerable multifunctional hydrogel (CuS_NPs_-HA-Fe^3+^-EDTA [EDTA: ethylenediaminetetraacetic acid disodium salt] hydrogel) termed CHFH through incorporating copper sulfide NPs (CuS_NPs_) with photothermal capability in the network of hyaluronate acid (HA) and Fe^3+^–EDTA complex. The hydrogel showed a strong affinity for bacteria and could accumulate them on its surface. The hyaluronidase (HAase) released by the bacteria then disintegrated the HA in CHFH, causing the Fe^3+^ release. In the bacterial microenvironment, the Fe^3+^ could be reduced to Fe^2+^ that could not only induce bacterial ferroptosis but also react with the endogenous H_2_O_2_ to generate •OH for CDT. Meanwhile, under NIR irradiation, the heat produced by the integrated CuS_NPs_ was transferred to the attached bacteria. The combination of PTT and localized CDT made it possible for bacterial killing at a relatively low temperature (45 °C), which could prevent the healthy tissue from thermal damage. The results of in vivo experiments demonstrated that the bacterial infection could be efficiently eliminated through PTT/CDT, which promoted the wound recovery and tissue reconstruction through reducing production of proinflammatory factors as well as increasing production of growth factors. Inspired by the excellent antibacterial effect of CHFH, the researchers then utilized CHFH to construct a Band-Aid for managing *S*. *aureus*-infected wound healing in vivo, and the satisfactory wound healing promotion effect of this Band-Aid confirmed the potential of the hydrogel for clinical application. In another study, Huang et al. [43] designed a light-activated injectable nanocomposite hydrogel. This system was based on Ag_2_S nanodots-conjugated Fe-doped bioactive glass NPs (BGN-Fe-Ag_2_S), 2,2′-azobis[2-(2-imidazolin-2-yl)propane]-dihydrochloride (AIPH), and poly(ethylene glycol) diacrylate (PEGDA) for cancer therapy, MDR bacteriostasis, and wound treatment (Fig. 4). In this hydrogel, Ag_2_S nanodots were utilized as the photothermal agent, which could generate hyperthermia under NIR laser irradiation to trigger the AIPH decomposition for the release of alkyl radicals, leading to PEGDA polymerization. During this process, the drug-containing liquid that was injected into the infected site could be gelatinized in situ, and the BGN-Fe-Ag_2_S was fixed in the lesion area. The overexpressed H_2_O_2_ in the inflammatory microenvironment could react with Fe^2+^ produced by hydrolyzed BGN-Fe for the generation of •OH. This in situ gelatinized hydrogel, with superb chemodynamic and photothermal activities, could not only kill MDR bacteria but also eliminate tumor during the treatment. Meanwhile, the hydrogel also expedited wound healing due to the hydrolysis of bioactive glass. 

In addition, some researchers used nanozymes to achieve antibacterial CDT. For example, Feng et al. [44] developed a mesoporous Fe-N-C single-atom nanozyme (SAzyme) with a uniform diameter, a high specific surface area, and a large pore size through a soft-template strategy for the antibacterial treatment. The mesoporous Fe-N-C SAzyme showed an outstanding photothermal conversion capability due to the carbon framework. Additionally, the Fe-N-C SAzyme also showed a high POD-mimicking activity and could convert H_2_O_2_ to highly toxic •OH that was able to damage the cell membrane and enhance the permeability and heat sensitivity of bacteria. Upon NIR light irradiation, the carbon framework could cause the temperature increase and thus enhance the POD-like catalytic activity of the SAzyme. Therefore, the Fe-N-C SAzyme exhibited a high antibacterial potency due to the combination of PTT and CDT. In vivo antibacterial experiments proved that the mesoporous SAzyme could avoid bacterial infection and accelerate wound healing. In addition, Xu and coworkers [45] developed a biofilm microenvironment (BME)-activatable Fe-doped polydiaminopyridine nanofusiform-mediated SAzyme (termed FePN SAzyme) for synergetic PTT/CDT of bacteria-infected wounds. The H_2_O_2_-activated photothermal conversion of SAzyme could be accelerated in the acidic inflammatory environment. The FePN SAzyme also achieved effective CDT resulting from the SAzyme-mediated Fenton reaction together with a high content of H_2_O_2_. Importantly, the FePN SAzyme could deplete GSH to overcome the biofilm resistance to oxidative stress and then improve the CDT effect. The SAzyme could also catalyze the decomposition of the BME-overexpressed H_2_O_2_ into O_2_, thus alleviating the biofilm hypoxia related to therapeutic resistance. Under 808-nm NIR irradiation, the FePN SAzyme effectively mediated synergistic PTT and CDT in vivo. In this system, on the one hand, the “off-on” switch of PTT property is controlled by H_2_O_2_ and H^+^ (in the BME) as well as exogenous NIR light stimulus, which could enhance the therapeutic accuracy and efficiency and minimize side effects; on the other hand, FePN SAzyme could also realize enhanced CDT by producing •OH via Fenton reaction and depleting GSH in the BME, thus helping to achieve a satisfactory anti-infection effect.

#### Combination of CDT and ST

**Fig. 5.**
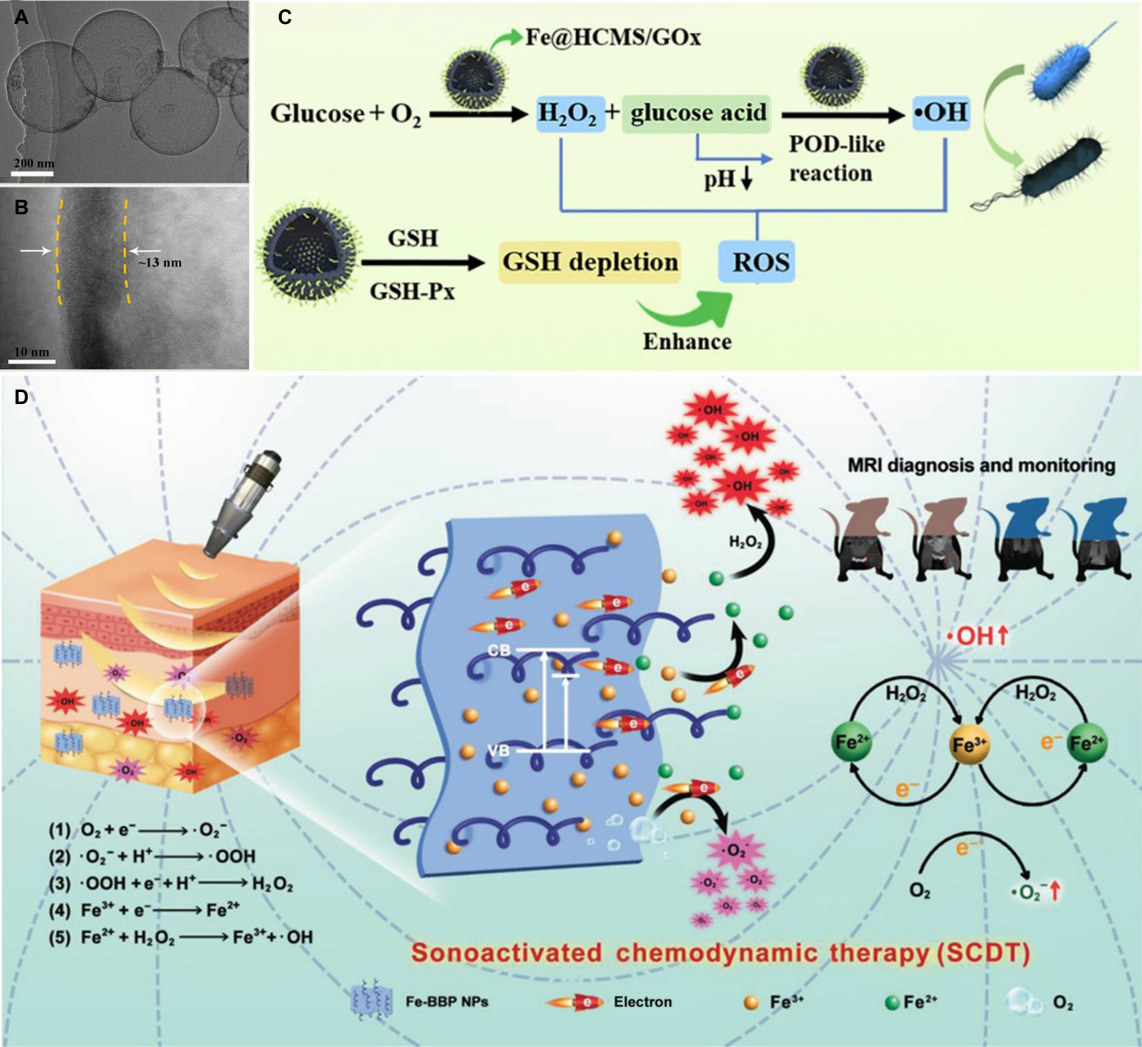
(A and B) Transmission electron microscopy images of Fe@HCMS. (C) Scheme showing the catalytic cascade activities of Fe@HCMS/GOx that can be utilized for bacterial inactivation. (A to C): Adapted from [58] with permission. Copyright 2022, Elsevier. (D) Schematic illustration of sonoactivated CDT mediated by Fe-BBP NPs against MRSA infection. Adapted from [67] with permission. Copyright 2020, Wiley-VCH.

Since the CDT efficiency is related to the concentration of H_2_O_2_, increasing the local H_2_O_2_ concentration is considered to be an ideal strategy to improve CDT efficacy. Glucose oxidase (GOx) is able to catalyze the reaction of oxygen and glucose to generate H_2_O_2_ and gluconic acid [57], and the generated H_2_O_2_ can meet the need of CDT and consume the glucose in bacteria for starvation therapy (ST). Therefore, synergistic CDT/ST has been adopted in several antibacterial systems [58–62]. For example, Zhang et al. [58] constructed a type of hybrid nanozyme (termed Fe@HCMS/GOx) with multienzyme-like activity through assembling GOx on a hollow mesoporous carbon nanosphere that was doped with single-atom Fe (Fig. 5A and B). GOx could catalyze the glucose in vivo to produce H_2_O_2_ that was then converted to toxic •OH by Fe@HCMS/GOx with Fe^3+^-mediated POD-mimicking activity for CDT. The GOx-catalyzed decomposition of glucose was also able to establish an environment with a low pH for the following POD-like reaction. Moreover, Fe@HCMS with GSH consumption capability could elevate the produced •OH level (Fig. 5C). The researchers then assembled the prepared Fe@HCMS/GOx on the polypropylene composite enveloped by bacterial cellulose to fabricate an antibacterial wound dressing. The in vivo experiments demonstrated that this kind of nanozyme-based wound dressing could effectively facilitate wound healing and had a great potential in CDT-mediated antibacterial treatment. In this work, the authors design a glucose-responsive nanozyme and assemble it on the dressing, providing a highly applicable CDT-based anti-infection strategy. Additionally, Li and coworkers [59] constructed a cascade catalytic nanoplatform for the antibacterial treatment. First, positively charged polymer capsules were obtained via the covalent self-assembly of laterally modified pillar[5]arenes (BDMP5) and methylation modification. Polymer capsules encapsulating Fe_3_O_4_ NPs (NCs/Fe_3_O_4_) were then prepared by covalent coassembly of a flexible linker and BDMP5. Finally, the authors constructed the GOx–NCs/Fe_3_O_4_ through the adsorption of negatively charged GOx on the surface of positively charged capsule. GOx could catalyze glucose into H_2_O_2_ and gluconic acid (thus realizing ST), which would trigger Fe_3_O_4_ NPs to release Fe^2+^. Subsequently, Fe^2+^ further catalyzed H_2_O_2_ into •OH through Fenton reaction. The generated •OH destroyed the bacterial membrane and caused the bacteria death, realizing chemodynamic antibacterial therapy. This work develops a GOx-assisted CDT based on Fe_3_O_4_ NPs for the bacterial treatment. Li et al. [60] designed a metal–organic framework (MOF)-based nanoreactor (termed ZIF-8@GOx@BHb) loaded with GOx and POD-like bovine hemoglobin (BHb) via pore encapsulation for combating MDR bacteria and promoting diabetic wound healing. On the one hand, GOx could deplete glucose and then cut off the nutrient supply of bacteria for ST. On the other hand, BHb, as an O_2_ carrier, was able to bring O_2_ to GOx. In addition, BHb, as a type of biocompatible protein, has a strong POD-like activity because of its Fe ion center. Therefore, the BHb converted the generated H_2_O_2_ into •OH for CDT. Together, ZIF-8@GOx@BHb exhibited an excellent killing effect against MDR bacteria. Moreover, the produced gluconic acid could cause an acidic environment, which was beneficial for both the elevation of POD-like activity and the suppression of the bacterial growth. Both in vitro and in vivo results confirmed that the designed nanoreactor could effectively combat MDR bacteria and promote the diabetic wound healing through synergistic CDT and ST, which may offer a new method for the infection treatment in diabetic patients.

#### Combination of CDT and SDT

Sonodynamic therapy (SDT), as an emerging ROS-generating therapeutic modality, is based on the combination of ultrasound (US), oxygen, and sonosensitizer [63,64]. The deep penetration of US in liquid media makes antimicrobial SDT a promising approach for treating deep-seated infections [65]. The synergistic application of CDT and SDT can improve the ROS yield, contributing to the better antibacterial effect [66,67]. Guo et al. [66] fabricated a Fenton reaction-reinforced antimicrobial SDT platform to treat *Enterococcus faecalis* (*E*. *faecalis*) infection in a root canal system. In this work, mesoporous silica NPs (MSNs) were first prepared. Next, MSNs were grafted with amino groups (MSN-NH_2_) and then conjugated with the sonosensitizer protoporphyrin IX to obtain M@P. Finally, Fe ions were anchored for yielding the final product termed M@P-Fe. Upon US irradiation, the loaded protoporphyrin IX was stimulated to yield ROS. Simultaneously, the Fe-mediated Fenton reaction further resulted in the •OH generation from H_2_O_2_. The authors demonstrated that this strategy combining SDT and CDT could efficiently eliminate *E*. *faecalis* infection and eradicate the *E*. *faecalis* biofilm under US irradiation without eliciting notable toxicity to MC3T3-E1 cells. Moreover, Song and coworkers [67] prepared a theranostic platform (termed Fe-BBP NPs) through grafting Fe^3+^ on the polyethylenimine-modified bismuth oxybromide (BiOBr) nanoplate for treating MDR bacterial infection (Fig. 5D). They found that the BiOBr NPs possessed sonocatalytic ability and could be activated by low-frequency US to generate ROS. Furthermore, the Fe^3+^ grafting could shorten the transport path of valence electrons as well as promote the electron (e^−^) and hole (h^+^) separation on the BiOBr NPs. With the US stimulation, oxygen molecules could easily capture excited electrons to produce O_2_^•−^, while Fe^3+^ trapped an interfacial charge to generate Fe^2+^ that could react with a high amount of H_2_O_2_ in an inflammation microenvironment to produce toxic •OH. Benefiting from the robust ROS production capacity and high tissue penetrability of US irradiation, the sonoactivated CDT mediated by Fe-BBP NPs can be used as a promising strategy for treating bacterial infections. More importantly, Fe^3+^ was also utilized for magnetic resonance imaging to obtain the accurate diagnosis of bacterial infection. In vitro and in vivo results showed that, with the assistance of US, Fe-BBP NPs could effectively eliminate MRSA. This work employs sonotriggered catalytic reactions for enhancing CDT efficiency and promoting ROS generation and provides a new solution to combat bacterial infection.

#### Combination of CDT and other therapies

Other therapeutic types have also been combined with Fe-based CDT for bacterial treatment [68–74].

For example, antimicrobial peptides (AMPs) are a type of short peptide molecules generated by most living creatures. AMPs can suppress or kill bacteria often through nonspecific mechanisms involving cell membrane damage and usually show broad-spectrum activity against parasitic microorganisms [75]. Therefore, the synergy of peptide–drug therapy and CDT can be a viable antimicrobial strategy. For example, considering the fact that linoleic acid hydroperoxide (LAHP) has been reported to react with Fe(II) for ^1^O_2_ generation via the Fenton-like reaction to achieve CDT [76], Huang and coworkers [68] developed a multifunctional nanoplatform for bacterial suppression. They designed a _D_(KLAK)_2_ peptide and conjugated linolic acid (which served as a hydrophobic moiety) to the N-terminus of the _D_(KLAK)_2_ peptide to initiate the molecular self-assembly. The linolic acid could then produce LAHP through lipid peroxidation. The final obtained product was termed LAOOH-OPA, which could self-assemble into NPs. The positively charged _D_(KLAK)_2_ peptide could rapidly bind with microbial cells through electrostatic interaction and subsequently damage the bacterial membrane via membrane insertion. When triggered by exogenous Fe^2+^, LAHP could produce ^1^O_2_ for achieving CDT, which elicited lipid bilayer leakage. Owing to the combined antibacterial mechanisms of CDT and AMP, this LAOOH-OPA effectively inhibited bacteria both in vitro and in vivo. The in vivo antibacterial effect in this work was achieved by intravenous injection of LAOOH-OPA and Fe^2+^. Integrating Fe^2+^ and LAOOH-OPA into a single type of NPs may be more suitable for practical clinical application.

Beside antibacterial drugs, there are also some antibacterial treatments achieved by physical damage. Physical damage is mechanically killing the bacteria through the structural properties of materials [77]. Generally, the target of physical damage is the bacterial surfaces, and the physicomechanical interactions between materials and bacterial surfaces result in bacterial killing and the inhibition of biofilm formation [77]. To achieve the dual physical/chemical antibacterial treatment, Liu et al. [69] employed catechin (as a reducing and capping agent), HAuCl_4_, and FeCl_3_ to produce gold NPs (GNPs) with Fe^3+^ chelation (termed pGNP-Fe). The pGNP-Fe could self-assemble on the bacteria because of the inherent attraction property of phenols, and then the physical pressure (due to the attachment of phenol-modified GNPs) caused bacterial membrane damage. The GNPs endowed pGNP-Fe with oxidase-like ability that could make use of O_2_ in the microenvironment to generate H_2_O_2_. Moreover, the chelation of Fe^3+^ on pGNP-Fe could stimulate the production of H_2_O_2_ and the chelated Fe^3+^ endowed the pGNP-Fe with POD-mimicking activity that was based on the Fe^3+^-mediated Fenton reaction, causing the production of massive ROS for CDT. A sufficient amount of H_2_O_2_ was produced steadily to catalytically generate ROS, consequently causing long-lasting and stable oxidative stress for bacterial killing. This work demonstrates that the synergistic physical/chemical interactions can endow pGNP-Fe with antibiotic-mimicking antibacterial property.

Bacteria-caused osteomyelitis occurs in deep bone tissues where NIR light cannot arrive, and thus, PTT is unable to achieve a good therapeutic effect. Microwave (MW) with a strong penetration capability was reported to be a satisfactory alternative for the treatment of deep tissue infections [70]. Wei et al. [70] designed a Na^+^-inserted Prussian blue (PB) system to serve as an MW-absorbing material and a carrier of Fe ions for treating bacteria-induced osteomyelitis. PB could absorb MW to produce heat because of the reflection of mesoporous structure and dielectric loss. The PB was excited by MW, which decreased the Fe(II)–(CN) and Fe(III)–(NC) bonds in PB. Then, Fe ions were released from the PB structure. PB, as a spin crossover structure material with the susceptibility to an electromagnetic field, promoted the change of the status of Fe ions inside PB from low-spin to high-spin. Moreover, Na^+^ insertion in PB accelerated the escape of Fe ions under MW radiation. MW irradiation could change the permeability of bacterial membrane, which allowed the released Fe^3+^ and Fe^2+^ to penetrate the membrane easily. Fe ions then reacted with H_2_O_2_ (through Fenton reaction) and GSH in the bacteria. Overall, the combination of MW, MW-mediated thermal effect, GSH consumption, and Fe^2+^ (or Fe^3+^)-induced Fenton reaction led to the bacterial death. This work successfully combines the MW-mediated thermal effect with CDT and provides an efficient method for treating deep bacterial infections.

As mentioned above, treating bacterial infections with antibiotics alone can lead to MDR bacteria. For achieving better antibacterial effects, researchers have combined antibiotic treatment with Fe-based CDT. For example, Wu and coworkers [74] constructed ampicillin (Amp)-loaded Fe^3+^-doped MOFs (termed nFMs@Amp) to treat H_2_O_2_-secreting *Streptococcus pneumoniae* (*S*. *pneumoniae*) infection. Fe^3+^ ions that were used as the metallic nodes in nFMs@Amp could react with bacteria-generated H_2_O_2_ and yield •OH through Fenton reaction (CDT effect). Therefore, the nFMs@Amp framework could break down and responsively release Amp in the presence of H_2_O_2_. Overall, nFMs@Amp could catalyze the decomposition of *S*. *pneumoniae*-secreted H_2_O_2_ and then protect the pulmonary tissue from H_2_O_2_-induced damage. •OH produced by Fe-mediated Fenton reaction showed the chemodynamic effect to eliminate drug-resistant bacteria, and the responsive release of Amp from the collapsed nFMs was used to kill drug-sensitive bacteria specifically. In in vivo experiments, nFMs@Amp killed more than 98% of *S*. *pneumoniae* and led to a survival rate of over 90% in the mice infected by a fatal dose of *S*. *pneumoniae*. This work provides a new avenue for the CDT-mediated treatment against H_2_O_2_-secreting bacteria.

#### CDT-involved multimodal therapy

To improve the therapeutic effects during bacterial treatments, many researchers have developed synergistic antibacterial systems which combine one or more treatment modalities with CDT [78–84]. For instance, Lai et al. [78] designed a multifunctional nanosystem (termed LL-37@MIL-101-Van) by covelently linking vancomycin to an MIL-101(Fe)–NH_2_ (abbreviated as MIL-101) (MIL: Material of Institut Lavoisier) core and then modifying the core with a targeting AMP LL-37 for the treatment of MRSA-caused infections. The authors demonstrated that the LL-37@MIL-101-Van composite NPs exhibited an accurate wound delivery capability due to the precise bacterial targeting mediated by LL-37. The MIL-101, which showed inherent POD-mimetic activity, responded to the acidic microenvironment and converted endogenous H_2_O_2_ to •OH via Fenton reaction for CDT. Together with the toxic •OH and the inherent antibacterial activities of vancomycin and LL-37, LL-37@MIL-101-Van exerted a combined antibacterial effect toward MRSA. Both in vitro and in vivo experiments proved that the specific targeting and elimination of MRSA were successfully realized. Overall, this work integrates antibacterial peptide, CDT, and antibiotic for targeted and efficient antibacterial treatment. Besides, Fu et al. [82] established a nanoplatform for multimodal antibacterial treatment. Specifically, GOx and indocyanine green (ICG) were loaded into zeolitic imidazolate framework-8 (ZIF-8), followed by the formation of a metal polyphenol network (MPN) consisting of Fe^3+^ and tannic acid. This nanoplatform was termed ZIF-ICG@ZIF-GOx@MPN. Under NIR light irradiation, the encapsulated ICG could produce both ^1^O_2_ and heat, realizing antibacterial PDT and PTT, respectively. Because of the existence of Fe ions, ZIF-ICG@ZIF-GOx@MPN could also produce •OH and achieve CDT. GOx could catalyze glucose (in the presence of O_2_ and H_2_O) into H_2_O_2_ and gluconic acid. The generated H_2_O_2_ acted as the substrate of the Fenton reaction and enhanced the CDT efficiency. The generated gluconic acid led to a pH reduction, which was beneficial to CDT. Besides, the heat produced in the ICG-mediated PTT process could also accelerate Fenton reaction and then enhance CDT. Overall, this nanoplatform realized a synergy of PDT, PTT, and triple (produced H_2_O_2_/reduced pH/generated heat)-enhanced CDT. Both in vitro and in vivo experimental results proved that this nanoplatform had superior antibacterial efficacy.

### Copper-mediated CDT

Copper ions play some important physiological roles and thus have attracted many researchers' attention. For example, it has been reported that they can enhance the proliferation of osteoblastic cells and vascularization [85]. In respect of the reactivity toward H_2_O_2_, Cu holds similar redox properties to Fe, that is, the reaction (referred to as Fenton-like reaction) between Cu^+^ and H_2_O_2_ can produce •OH and Cu^2+^ [86]. Importantly, Cu is considered to be a better choice for CDT, because the Cu^+^/H_2_O_2_ system can produce •OH over a broader pH range, while the Fe^2+^/H_2_O_2_ system can work only under acidic conditions [86].

#### Single CDT

**Fig. 6.**
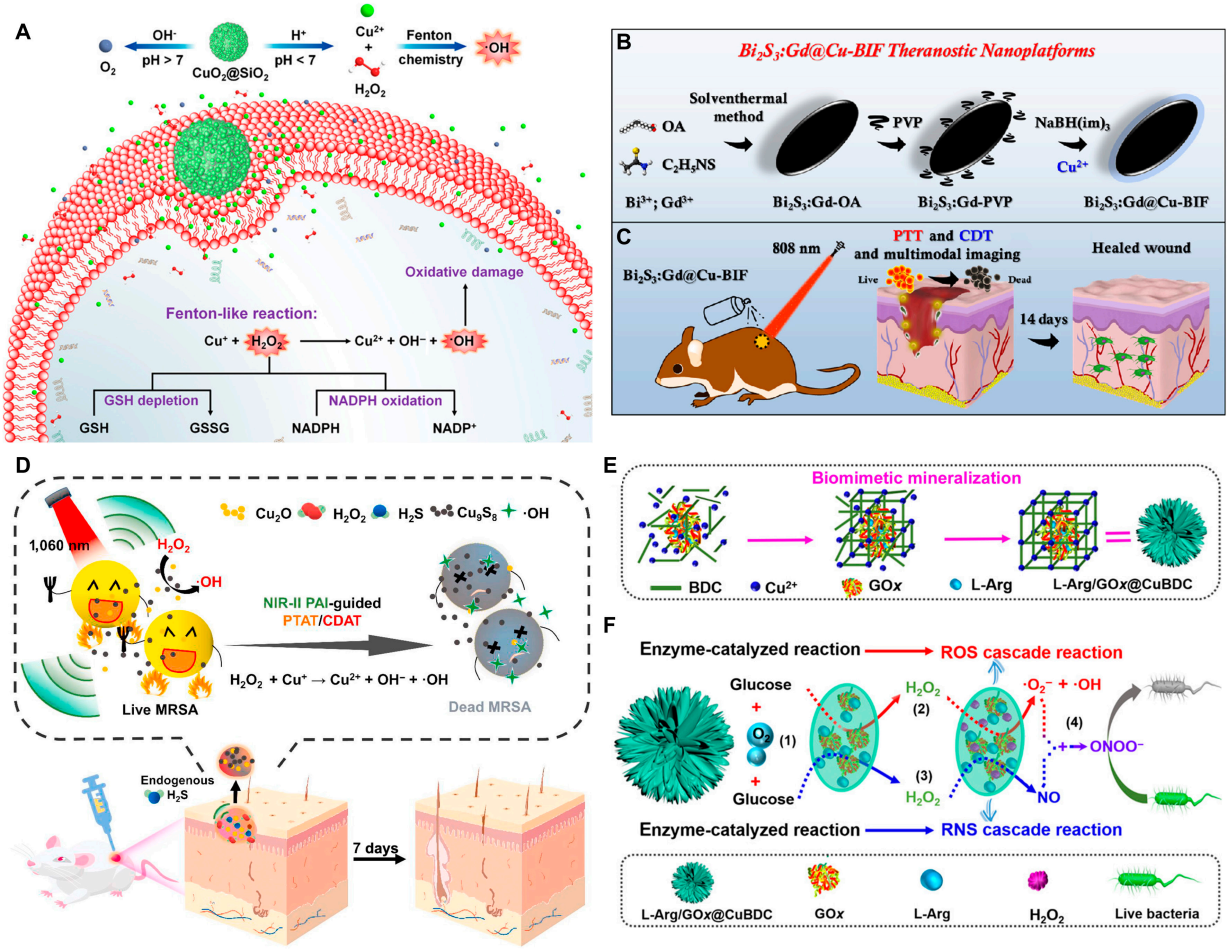
(A) Illustration showing the working mechanism of CuO_2_@SiO_2_ for ROS generation, H_2_O_2_ self-supply, and in situ O_2_ production in bacterial cells. Adapted from [87] with permission. Copyright 2021, American Chemical Society. Schemes illustrating (B) the construction procedure of the Bi_2_S_3_:Gd@Cu-BIF nanoassembly and (C) the working mechanism of the nanoassembly for synergistic PTT/CDT. (B and C): Adapted from [107] with permission. Copyright 2022, Elsevier. (D) Scheme of the Cu_2_O NPs-mediated photothermal/chemodynamic synergistic anti-infection therapy. Adapted from [108] with permission. Copyright 2021, Elsevier. (E) Synthetic process of L-Arg/GOx@CuBDC by biomimetic mineralization and (F) the reaction mechanism for synergistic bacterial killing. (E and F): Adapted from [121] with permission. Copyright 2020, American Chemical Society.

Considering the properties of Cu ions mentioned above, researchers have developed a variety of Cu-containing materials bearing POD-like activity to realize antibacterial CDT [87–102]. For instance, Li et al. [87] constructed a pH-responsive Fenton-like nanoplatform through assembling copper peroxide (CuO_2_) nanodots in pomegranate-like mesoporous silica nanoshells (Fig. 6A). Specifically, the CuO_2_ nanodots were protected by PVP and further encapsulated in mesoporous silica nanoshells to obtain CuO_2_@SiO_2_. In the acidic environment, the decomposition of CuO_2_@SiO_2_ produced Fenton catalytic Cu^2+^ ions and H_2_O_2_ to allow the pH-dependent •OH generation, while in a neutral medium (pH = 7.4), the controllable and prolonged O_2_ evolution occurred. The CuO_2_@SiO_2_ was capable of supplying H_2_O_2_ for Cu-mediated CDT, and in vitro as well as in vivo data demonstrated that this nanocomposite exhibited a boosted antibacterial capability and could accelerate wound healing. The use of CuO_2_ overcomes the H_2_O_2_ shortage in bacteria, and such a Cu-based CDT agent can also be applied in other CDT-involved antibacterial treatments. Additionally, Wang and coworkers [88] prepared 2-dimensional (2D) Cu-MOF NSs composed of 2-methylimidazoie and Cu ions via a simple one-step method. The 2D Cu-MOF NSs exhibited POD-like activity, which might be due to the high-density Cu^2+^/Cu^+^ on the surface, and could convert H_2_O_2_ into highly cytotoxic •OH in situ. Moreover, the high-density active sites and the large specific surface area enabled Cu-MOF NSs to interact with the bacterial surface and directly damage the lipids and proteins on the bacterial surface, thereby inducing a synergistic antibacterial effect together with CDT. In vitro experimental results proved that the 2D Cu-MOF NSs showed a satisfactory antibacterial effect against *S*. *aureus* and could eradicate the *S*. *aureus* biofilm. This work demonstrates the potential of Cu-MOF NSs as POD mimics in ROS-mediated antibacterial treatments. Gao et al. [89] utilized an ionic liquid (IL) to develop a Cu^2+^-loaded ionic gel (termed D-IL-Gel) for antibacterial and wound healing purposes. Dopamine polymerized along a polyvinyl alcohol chain was used as a continuous phase of the ionic gel. Cu^2+^ ions coordinated with PDA were incorporated into the polyvinyl alcohol/PDA polymer network. The IL choline–glycolate facilitated the gel formation via the hydrogen bonding interaction with polymer chains. The authors demonstrated that the D-IL-Gel had outstanding CDT activity and generated •OH effectively by Cu^2+^-driven Fenton-like reaction. In vivo data showed that with the assistance of a small amount of H_2_O_2_, the CDT activity of the D-IL-Gel could achieve efficient antibacterial performance. Moreover, choline–glycolate endowed the ionic gel with transdermal ability, thus enhancing the transdermal delivery of Cu^2+^, which was demonstrated to stimulate cell migration and contribute to wound reconstruction. Besides, Sun et al. [90] prepared chitosan-copper-gallic acid nanocomposites (CS-Cu-GA NCs) with both oxidase- and POD-like activities. The authors confirmed that the physiologically relevant antioxidants (AH_2_) in bacteria could be oxidized to generate H_2_O_2_ and A (in the presence of O_2_) when the oxidase-like CS-Cu-GA NCs interacted with bacteria. Then, the generated H_2_O_2_ was further catalyzed by the POD-like CS-Cu-GA NCs to produce •OH for effective antibacterial CDT. Additionally, CS-Cu-GA NCs integrated the innate antibacterial abilities of Cu NPs, chitosan, and Cu^2+^, which have been reported previously [103–105]. For clinical application, the chitosan liquid gel containing CS-Cu-GA NCs was fabricated into a Band-Aid, and the authors prepared a portable antibacterial product through fixing this Band-Aid in a medical adhesive tape. This work proposes a new solution for the preparation of antibacterial products with the function of multiple mechanisms-mediated synergistic sterilization. Finally, He and coworkers [91] prepared a Cu_2_O@CuO nanozyme by employing ascorbic acid (AA) as a protecting and reducing agent in an alkaline solution. Then, they added GSH to further etch the obtained Cu_2_O@CuO nanodots to construct Cu@Cu_2_S nanodots. Compared with Cu_2_O@CuO, Cu@Cu_2_S had more Cu(I) which had a high activity of Fenton-like reaction. The Cu@Cu_2_S nanodots thus exhibited higher POD-mimicking, oxidase-mimicking, and antibacterial activities. Furthermore, Cu@Cu_2_S showed strong red fluorescence for fluorescence imaging, which was used for visualizing the antibacterial process. Fluorescence imaging and scanning electron microscopy indicated that the Cu@Cu_2_S nanozyme could bind to the bacterial surface, destroy the cell wall, and finally lead to bacterial death.

#### Combination of CDT and PTT

Similar to the Fe-involved antibacterial CDT, the heat produced in PTT process is able to promote Cu-mediated Fenton-like reaction, thus elevating the efficacy of Cu-based CDT [106–114]. For example, Wang et al. [106] constructed Cu single-atom sites/N-doped porous carbon (named as Cu SASs/NPC) for antibacterial therapy. The Cu SASs/NPC showed POD-like activity in the presence of H_2_O_2_, thus producing abundant •OH (the CDT effect) to kill bacteria and make bacteria more susceptible to temperature. Furthermore, the Cu SASs/NPC had excellent NIR light absorption. Under NIR light irradiation, the Cu SASs/NPC could transform light energy into heat energy that could kill bacteria and enhance the POD-mimicking activity. At mild temperatures, the increased component movement caused the increase in the destructive physical interaction of the sharp edges of Cu SASs/NPC with bacteria. Interestingly, Cu SASs/NPC could consume GSH in bacteria, thereby remarkably enhancing the effect of the ROS-mediated therapy. In vivo results exhibited that Cu SASs/NPC could effectively eradicate infections caused by MRSA and thus achieve promoted wound healing. This work provides a good example of the application of Cu single-atom-containing catalysts in antibacterial CDT. In another study, Qi and coworkers [107] synthesized a Gd-doped Bi_2_S_3_@Cu(II) boron imidazolate framework (termed Bi_2_S_3_:Gd@Cu-BIF) nanoassembly for the healing of the wounds infected by MRSA (Fig. 6B and C). The Bi_2_S_3_:Gd@Cu-BIF nanoassembly exhibited an outstanding photothermal conversion ability under 808-nm NIR laser irradiation. In the inflammatory microenvironment, Cu^2+^ and Cu^+^ ions could be released from Cu-BIF. Cu^2+^ then reacted with GSH and generated Cu^+^ which could donate free electrons through intracellular Fenton-like reaction and Russel mechanism to generate •OH and ^1^O_2_, respectively. The possible reactions were described by the following equations: (1) Cu^2+^ + GSH → Cu^+^ + GSSG; (2) Cu^+^ + H_2_O_2_ → OH^−^ + Cu^2+^ + •OH; (3) Cu^+^ + H_2_O + O_2_ → H_2_O_2_ + Cu^2+^ + ^1^O_2_ + OH^−^. Besides ROS generation, the nanoassembly could also disturb the redox equilibria in bacteria through GSH consumption. The in vivo results proved that this synergistic CDT/PTT could facilitate endothelial cell angiogenesis as well as fibroblast migration, leading to the acceleration of wound healing. Furthermore, Gd^3+^ and Bi^3+^ in Bi_2_S_3_:Gd@Cu-BIF were employed as magnetic resonance imaging and computed tomography contrast agents for the accurate diagnosis of MRSA-infected abscess. Additionally, Yang et al. [108] prepared a theranostic platform based on Cu_2_O NPs for photothermal and chemodynamic antibacterial therapy (Fig. 6D). Cu_2_O NPs exhibited high susceptibility to H_2_O_2_ and H_2_S in the infection microenvironment. After in situ injection, Cu_2_O NPs rapidly reacted with the endogenously overexpressed H_2_S to produce Cu_9_S_8_ NPs in the inflammatory region. The produced Cu_9_S_8_ with high NIR-II light absorption capacity could be utilized for H_2_S-activated PTT and serve as an NIR-II photoacoustic imaging agent under 1,060-nm laser irradiation. Moreover, Cu_2_O NPs effectively catalyzed endogenous H_2_O_2_ to produce cytotoxic •OH via a Fenton-like reaction (CDT effect). Both in vitro and in vivo experiments proved that Cu_2_O NPs quickly destroyed most of the MRSA bacteria through infection microenvironment-activated PTT and obtained an effective and continuous elimination of the remaining bacteria through the •OH production that was enhanced by the PTT-caused heat, thus realizing a synergistic antibacterial effect. At the same time, the released Cu_2_O NPs were also able to promote the reepithelialization of the infected skin. This work develops an infection microenvironment-activated antibacterial strategy with reduced side effects based on Cu_2_O NPs that are synthesized through a fast, simple, and low-cost method. Niu et al. [109] developed a light-responsive drug-delivery hybrid hydrogel for synergistic photothermal-chemodynamic therapy. Specifically, a dark blue charge-transfer complex (termed TMC) was generated via simply mixing CuCl_2_ and 3,3′,5,5′-tetramethylbenzidine. The formed TMC exhibited strong NIR-II light absorption and superb photothermal capability. Next, a low-melting point agar was used as the carrier of TMC, and a certain amount of H_2_O_2_ was added into the gel to obtain TMH@Gel. Upon light irradiation, TMH@Gel effectively converted light energy into heat energy and could serve as an excellent photothermal agent. Moreover, the generated heat softened and melted the agar, thus releasing the Cu^2+^ and H_2_O_2_ into the bacterial environment. The Fenton-like reaction between H_2_O_2_ and Cu^2+^ was improved by the photothermal effect, and the toxic •OH was generated to further kill bacteria. The excellent killing capacity of TMH@Gel toward *S*. *aureus* and *E*. *coli* was demonstrated in vitro. In addition, in vivo data showed that TMH@Gel plus NIR-II laser irradiation could accelerate wound healing in the *S*. *aureus*-infected mice, which proved the potential of TMH@Gel in antimicrobial application.

#### Combination of CDT and other therapies

Apart from the abovementioned synergy of PTT and CDT, Cu-based CDT has also been combined with other therapies for antibacterial purposes [115–120]. For example, Peng et al. [115] proposed a bimetal MOF domino microreactor (named as BMOF-DMR) composed of GOx and Cu-doped ZIF-8 for synergistic antibacterial ST/CDT. Specifically, GOx first consumed glucose to suppress bacterial metabolism (achieving ST) and generated H_2_O_2_ and gluconic acid through an enzymatic reaction. In the acidic environment, BMOF-DMRs could degrade, along with a slow release of Zn^2+^ and Cu^2+^. The released Cu^2+^ could activate Fenton-like reaction for •OH generation (achieving CDT), and the released Zn^2+^ and •OH further invaded bacterial cells and inactivated the DNA and enzymes of bacteria for robust sterilization. Moreover, owing to the release of Cu^2+^, the upregulated expression of vascular endothelial growth factor and in vivo angiogenesis were found in the BMOF-DMR-mediated antibacterial treatment, which was demonstrated in the in vitro and in vivo assessments. In addition, Liang et al. [120] developed a CuO_2_/TiO_2_ heterostructure composed of oxygen vacancy-rich porous titanium oxide (OV-TiO_2_) and CuO_2_ nanoclusters for sonothermal and sono-chemodynamic antibacterial therapy. The CuO_2_ on the surface of sonosensitized OV-TiO_2_ could (a) endow the material with Fenton catalytic ability for CDT and (b) enhance the sonothermal and sonodynamic performance of TiO_2_ via narrowing the TiO_2_ bandgap and reducing the recombination rate of electron–hole pairs. For further application, the authors then built a CuO_2_/TiO_2_ integrated microneedle (CTMN) patch, which could deliver nanotherapeutics to the deep dermis to achieve sonothermal and sono-chemodynamic antibacterial therapy under US. The high antibacterial efficacy of this CTMN patch with the help of US was demonstrated by the high elimination rate (>99.9999%) against MRSA and *Pseudomonas aeruginosa* (*P*. *aeruginosa*) in vitro and the accelerated wound healing in vivo.

#### CDT-involved multimodal therapy

Moreover, there are also some nanoplatforms capable of realizing CDT-involved multimodal therapies [85,121–126]. Different types of therapies are combined to meet different treatment requirements and realize desired antibacterial therapeutic effects. Cheng et al. [121] fabricated a MOF-based therapeutic platform for synergistic bacterial killing (Fig. 6E and F). This biomimetic multienzyme system (termed L-Arg/GOx@CuBDC) was formed through the coencapsulation of L-arginine (L-Arg) and GOx into the urchin-like Cu-MOF. During the L-Arg/GOx@CuBDC-mediated reaction process, the glucose oxidation catalyzed by GOx was the initiation reaction, yielding H_2_O_2_ and gluconic acid. Next, on one hand, the produced H_2_O_2_ was converted into toxic O_2_^•−^ and •OH through the Fenton-like reaction mediated by Cu-MOF for CDT; on the other hand, the H_2_O_2_ oxidized L-Arg into NO (for gas therapy), and NO then quickly reacted with O_2_^•−^ to generate ONOO^−^, which was more cytotoxic than NO or O_2_^•−^. Taken together, the L-Arg/GOx@CuBDC showed a robust bactericidal function (in vitro bacterial inactivation rate: ≥97%) due to the formed ROS, ONOO^−^, and NO. This work proposes an effective antibacterial strategy that combines CDT with ST and gas therapy, which may have implications for future related research. Additionally, Lu et al. [122] developed a CS-based electrospun nanofiber (ENF) material (termed ENFC) via modifying the CS ENF membrane with fucoidan (Fu) and CuS NPs for bacterial killing and bone tissue engineering applications. CuS NPs served as a photothermal and chemodynamic material, which could kill bacteria through the hyperthermia effect and toxic •OH generated in Fenton-like reaction, respectively. In addition, the ENFC with photocatalytic activity could generate ROS under NIR light irradiation for achieving the photodynamic bactericidal effect. In vitro results showed that the ENFC + H_2_O_2_ + NIR system exhibited a satisfactory antibacterial effect against *S*. *aureus* and *E*. *coli* because of the photothermal and chemo-photodynamic effects. Moreover, Fu and Cu ions could be released from the ENFC under acidic inflamed tissues and facilitate the capillary tube construction of endothelial cells and enhance the alkaline phosphatase activity of osteoblast cells. This work proposes a new method for preventing bone infection and promoting tissue regeneration.

### Manganese-mediated CDT

**Fig. 7.**
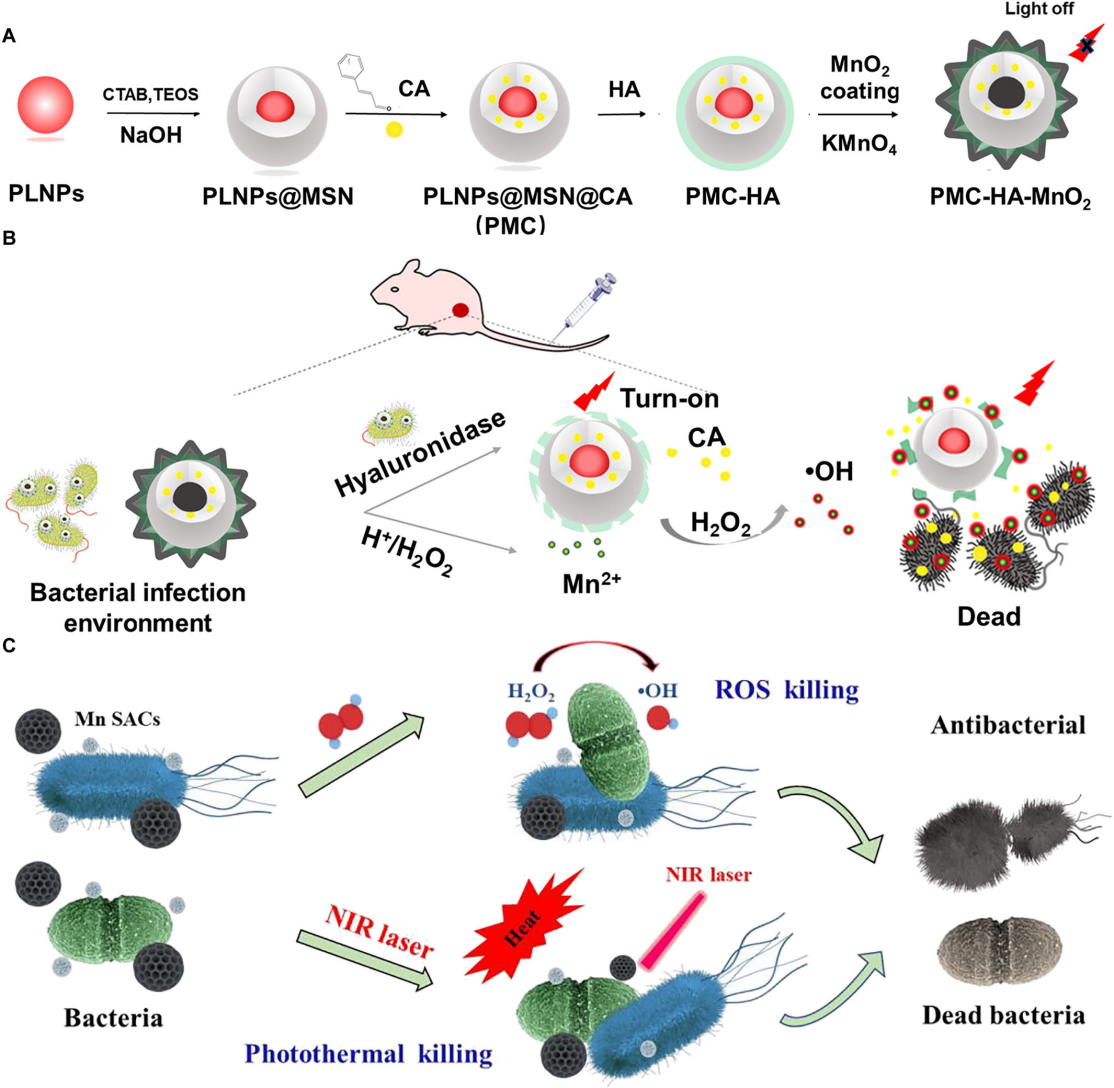
(A) Scheme showing the preparation process of PMC-HA-MnO_2_. (B) Working mechanisms of PMC-HA-MnO_2_ for bacterial microenvironment-responsive PL imaging, HAase-activated CA release, and CDT. (A and B): Adapted from [129] with permission. Copyright 2022, Elsevier. (C) Antibacterial mechanism of Mn SACs for CDT/ PTT. Adapted from [130] with permission. Copyright 2022, Elsevier.

Unlike Fe and Cu, Mn is able to exist in a wider range of oxidation states (from 0 to +7) [86]. The Fenton-like reaction mediated by Mn in the presence of HCO_3_^−^ and H_2_O_2_ can be realized due to the interconversion between Mn^2+^ and Mn^4+^ [86,127]. Accordingly, Mn-based materials have been used for CDT-involved antibacterial treatments [128–136]. For example, in the study of Li et al. [128], through decorating manganese oxide (MnO*_x_*) NSs on the silicon nanowire array (SN) surface deposited with PDA, the authors prepared SN@PDA@MnO*_x_* for antibacterial therapy. The SN with huge specific surface area strongly supported the adhesion of dopamine as well as the dispersion of MnO*_x_*. Since Mn^2+^ can be released from MnO_2_, which is the active component in MnO*_x_* [137,138], Mn^2+^ and MnO_2_ from SN@PDA@MnO*_x_* had a combination effect for generating ROS via a chemodynamic reaction. Besides, MnO*_x_* could be reduced by PDA and then dissolved in the solution, releasing a trace amount of Mn ions for catalyzing the production of ROS. At the same time, the H_2_O_2_ produced by PDA in the self-circulating redox process might be one of the reasons why SN@PDA@MnO*_x_* could produce ROS. In in vitro studies, SN@PDA@MnO*_x_* could release a large number of ROS within 2 h, which could achieve a 99.99% antibacterial effect, demonstrating the potential of SN@PDA@MnO*_x_* for antibacterial application. In addition, Wang and coworkers [129] reported a nanosystem that could be triggered by the bacterial microenvironment to achieve bacterial elimination and persistent luminescence (PL) “turn-on” imaging (Fig. 7A and B). Specifically, the mesoporous silica-coated PL NPs (PLNPs@MSN) were loaded with CA that had a broad bactericidal ability. Then, hyaluronic acid (HA) was then grafted on PLNPs@MSN@CA. Finally, the in situ growth of MnO_2_ shells resulted in the formation of PLNPs@MSN@CA-HA-MnO_2_ (named as PMC-HA-MnO_2_). The PMC-HA-MnO_2_ could accumulate in the bacterial infection site with a low pH because of the protonation of the amino group. Then, the MnO_2_ shell disintegrated due to the acidic environment and the overproduced H_2_O_2_ in the infection environment. Therefore, the PL of PLNPs was activated, and the generated Mn^2+^ from MnO_2_ decomposition served as a Fenton-like reagent that could produce •OH for CDT. The infected region was illuminated by the restored PL for monitoring the treatment process. In the meantime, HA was decomposed by HAase secreted by bacteria, and then the encapsulated CA was released for directly killing bacteria. Further, the antibacterial platform was successfully applied to treat MRSA-infected mice without eliciting damage to normal tissues. Overall, this multifunctional nanoplatform holds the potential of realizing the visualized treatment of MDR bacteria. In another example, Xu et al. [130] developed spherical mesoporous manganese single-atom catalysts (termed Mn SACs) based on dopamine hydrochloride and Pluronic F127 using a nanoemulsion assembly approach for photothermal-chemodynamic antibacterial therapy (Fig. 7C). The Mn SACs with outstanding POD-mimicking activity could generate a large amount of •OH in the presence of H_2_O_2_ via Fenton-like reaction. Besides, the photothermal conversion capacity of Mn SACs under NIR light irradiation made Mn SACs yield heat to inactivate bacteria and promote the CDT process to generate more ROS to kill bacteria. In vivo results exhibited that Mn SACs could avoid early inflammatory responses in the infected regions and promote the palingenetic angiogenesis, realizing a better wound healing effect. This study proposes a synergistic CDT/PTT antibacterial strategy based on Mn SACs. Li et al. [134] construct a nanogenerator (termed MnO_2_/GOx/AIBI) through loading a thermal-labile azo initiator (2,2'-azobis[2-(2-imidazolin-2-yl)propane]dihydrochloride, abbreviated as AIBI) and GOx on the flower-like MnO_2_ for bacterial infection management. In this system, the MnO_2_ nanoflower possessing the catalase-like activity could catalyze H_2_O_2_ to O_2_, which relieved hypoxia in the infection regions, while the loaded GOx could catalyze the reaction of O_2_, H_2_O, and glucose to produce H_2_O_2_ and gluconic acid, both of which triggered the degradation of MnO_2_ to form O_2_ and Mn^2+^. Then, the released Mn^2+^ could react with H_2_O_2_ and produce •OH via Fenton-like reaction to achieve CDT. Besides, MnO_2_ with superb NIR light absorption and photothermal conversion performance could induce local photonic hyperpyrexia that activated the decomposition of AIBI for •R production, thereby achieving photothermal dynamic therapy. Finally, the authors verified that the MnO_2_/GOx/AIBI showed excellent antibacterial effects both in vitro and in vivo because of the synergistic CDT/photothermal dynamic therapy.

### CDT based on other metal elements (Co, Mo, Pt, W, Ni, Ag, Ru, V, Au, and Zn)

Besides the commonly used metal elements (e.g., Fe, Cu, and Mn), other metal elements that have multiple redox states can also convert H_2_O_2_ into toxic •OH via Fenton-like reactions for antibacterial CDT. Recently, metal elements like cobalt (Co) [139,140], molybdenum (Mo) [141–145], platinum (Pt) [146,147], tungsten (W) [148,149], nickel (Ni) [150,151], silver (Ag) [152,153], ruthenium (Ru) [154], vanadium (V) [155,156], gold (Au) [157], and zinc (Zn) [158] have been applied to the design of antibacterial platforms.

Co^2+^, as a Fenton-like catalyst, has been widely studied for the oxidation of organic contaminants [86]. Furthermore, Co^2+^ can also be employed for CDT-involved antibacterial treatments [139,140]. For example, Ma and coworkers [139] designed a pH-responsive CaO_2_/GQDs@ZIF-67 composite nanosystem with H_2_O_2_ and O_2_ self-supplying ability for CDT/PDT of wound infection. In the acidic inflammatory condition, ZIF-67 could degrade to generate Co^2+^ and release CaO_2_ as well as graphene quantum dots (GQDs). The exposed CaO_2_ was able to react with water to produce H_2_O_2_ and O_2_. With the light-emitting diode irradiation, the GQDs could generate ^1^O_2_ for PDT, which could be enhanced by the self-supplied O_2_. The O_2_ also alleviated hypoxia at the site of inflammation. Moreover, the produced H_2_O_2_ could be catalyzed by Co^2+^ to produce •OH for Co^2+^-triggered CDT. The ROS produced by CaO_2_/GQDs@ZIF-67 could destroy the bacterial structure, lead to serious membrane damage and RNA and DNA leakage from the cytoplasm, and finally cause cell death. The combined PDT/CDT effect of CaO_2_/GQDs@ZIF-67 against *S*. *aureus* and *E*. *coli* was confirmed in in vitro and in vivo experiments. The authors also found that CaO_2_/GQDs@ZIF-67 might activate the immune response and then improve the therapeutic effect of the drug. Overall, this research has proved the feasibility of employing Co-containing materials for antibacterial CDT.

Mo has also been used for chemodynamic antibacterial therapy [141–145]. Shi et al. [141] developed an acidity-responsive polyoxometalate (POM) based on W/Mo for photothermal effect-enhanced CDT. The POM could self-assemble into larger-sized aggregates, allowing it to reside in the acidic infected site. Then, the H_2_O_2_ in the infected region could be catalyzed to produce •OH by the Mo^5+^ from the POM via the Fenton-like reaction. Meanwhile, Mo^5+^ was oxidized to Mo^6+^, and the latter was then reduced to Mo^5+^ by GSH to consume GSH and enhance the CDT effect. Moreover, the aggregated POM showed a stronger NIR light absorption capacity and could thus lead to the photothermal toxicity toward bacteria as well as realize photothermal effect-improved CDT of bacteria under 1,060-nm laser irradiation. In in vivo results, the POM with inflammatory retention had an outstanding chemodynamic/photothermal therapeutic effect, leading to the efficient eradication of *S*. *aureus*.

**Fig. 8.**
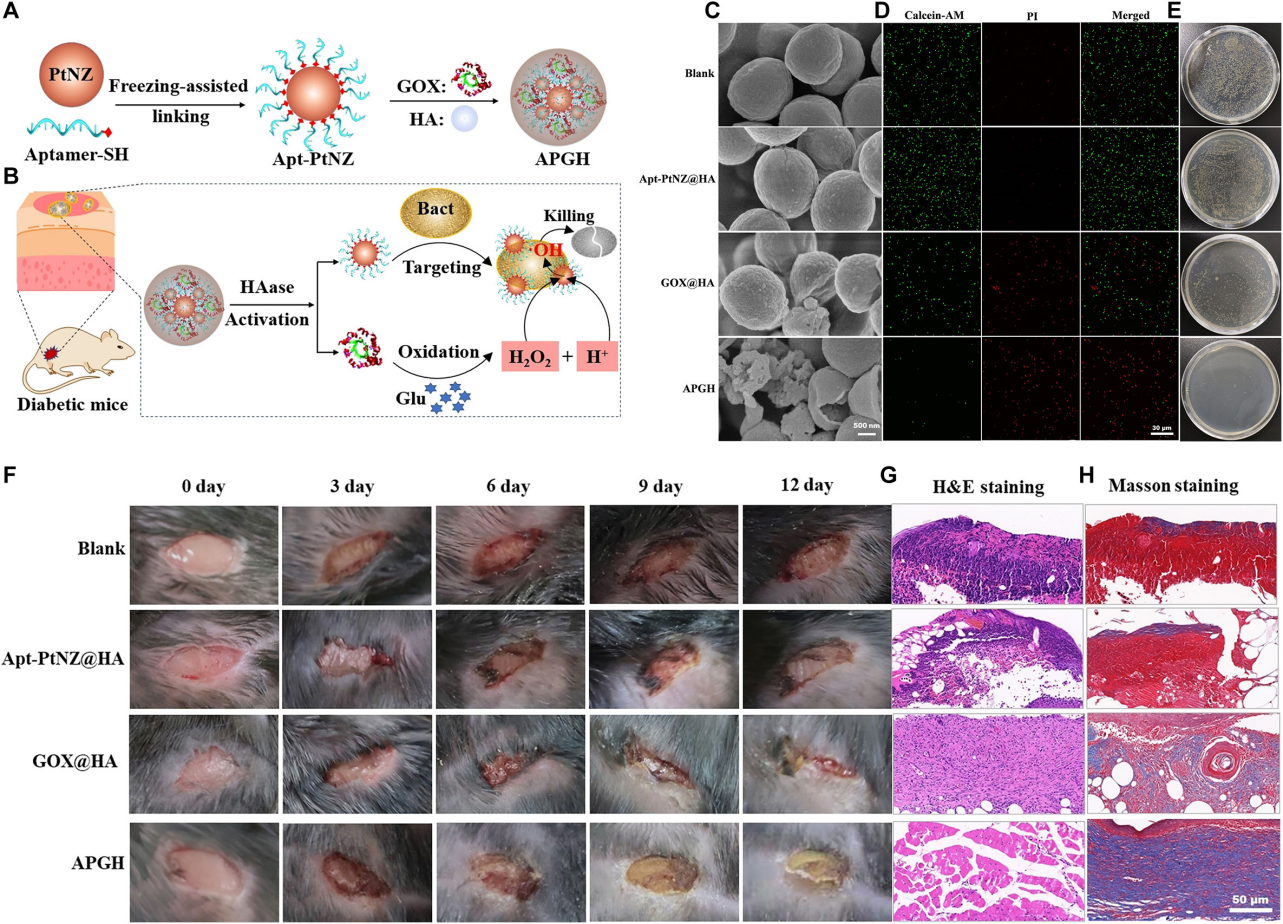
(A) Preparation route of the nanozyme capsule APGH and (B) its application for chemodynamic sterilization. (C) Scanning electron microscopy images, (D) calcein-AM/PI-stained fluorescence images, and (E) bacterial colony photographs of *S*. *aureus* subjected to different treatments. (F) Photographs of the wounds that were infected by *S*. *aureus* in the diabetic mice after various treatments for different days. (G) Hematoxylin and eosin (H&E) and (H) Masson staining images of the infected tissues after being treated by different materials for 12 days. (A to H): Adapted from [146] with permission. Copyright 2021, Wiley-VCH.

Some strategies have been designed for realizing Pt-mediated CDT [146,147]. For instance, Chen et al. [146] reported an activatable nanozyme with targeting ability for CDT (Fig. 8A and B). The aptamer-modified platinum nanozyme (Apt-PtNZ) and GOx were cocaged in an HA shell to obtain a nanocapsule termed APGH. The APGH was utilized for treating bacterial infection in a diabetic wound that featured an alkaline pH and a high level of glucose. After the addition of APGH to the wound, the HA shell was firstly decomposed by bacteria-secreted HAase, leading to the release of GOx and Apt-PtNZ. Next, the Apt-PtNZ could target bacteria selectively via aptamer binding. In the presence of H_2_O_2_, the Apt-PtNZ with a POD-like activity could generate •OH for CDT, while GOx could catalyze glucose (together with O_2_ and H_2_O) to yield H_2_O_2_ and gluconic acid. The produced gluconic acid lowered the pH in the wound site to promote the POD-like catalytic activity, and the generated H_2_O_2_ was used for in situ production of •OH on the bacterial surface. The antibacterial effect was verified both in vitro (Fig. 8C to E) and in vivo (Fig. 8F to H). This work utilizes GOx to break the H_2_O_2_ and pH limitations of CDT for diabetic wound healing. Moreover, owing to the aptamer-mediated targeting function of Apt-PtNZ, the in situ generation of •OH was realized, which solved the problem of the short lifetime of •OH, resulting in enhanced chemodynamic sterilization. Overall, this work achieves the accurate and improved CDT through sequential HA shell degradation and aptamer binding.

**Fig. 9.**
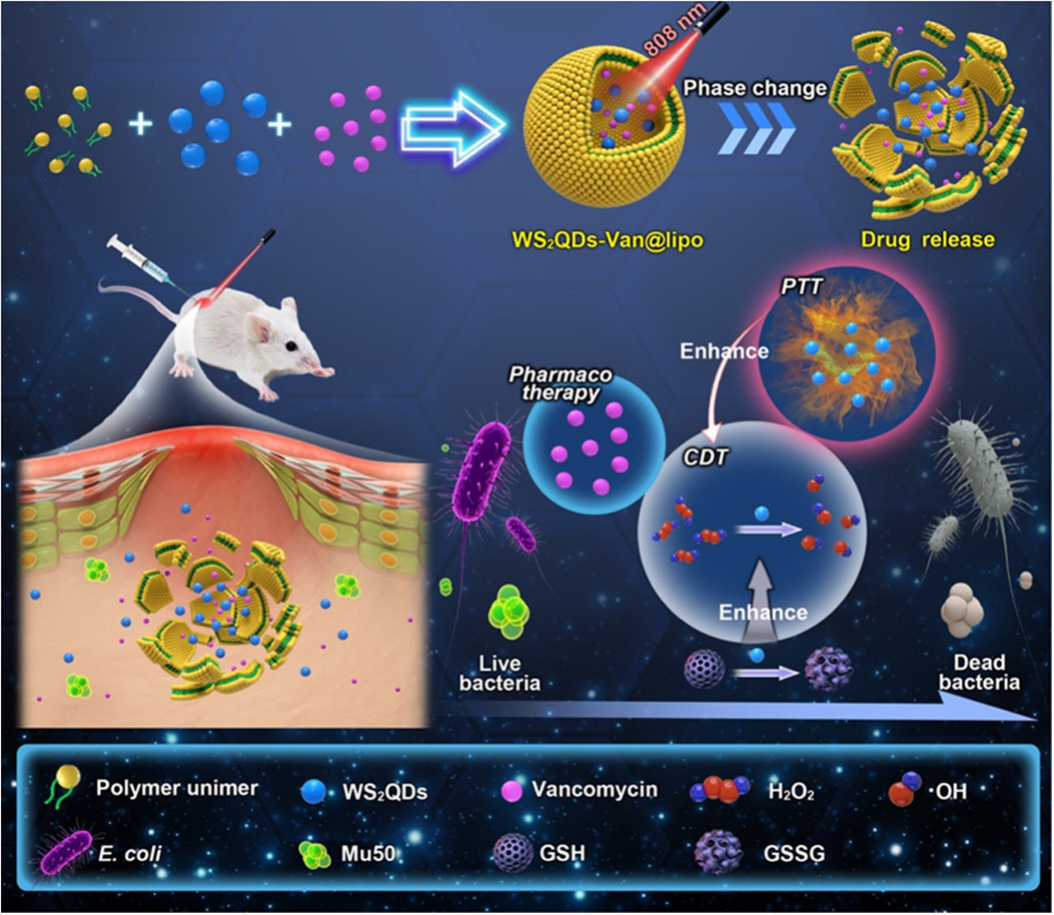
Scheme illustrating the synergistic PTT/CDT/pharmaco therapy that adopts WS_2_QDs-Van@lipo to treat the MDR bacteria-caused infection. Adapted from [148] with permission. Copyright 2020, American Chemical Society.

W-based antibacterial CDT has also been developed [148,149]. For example, Xu et al. [148] developed an NIR light-controlled antibacterial nanosystem (WS_2_QDs-Van@lipo) that was fabricated by encapsulating the antibiotic vancomycin and the tungsten sulfide quantum dots (WS_2_QDs) in a thermosensitive liposome (Fig. 9). Because of the photothermal conversion capability of WS_2_QDs, the thermosensitive liposomes could be damaged under NIR laser irradiation, causing the precise release of drugs. The WS_2_QDs exhibited a POD-mimicking activity in the presence of H_2_O_2_, which catalyzed H_2_O_2_ to •OH and conferred an excellent antibacterial CDT effect. Furthermore, the WS_2_QDs with the oxidase-mimicking activity also achieved superb GSH consumption, restraining ROS depletion and hence enhancing CDT efficacy. The photothermal effect of WS_2_QDs could potentiate the CDT effect to boost the antibacterial effect. The released vancomycin also synergistically eradicated bacteria. Overall, by combining WS_2_QDs possessing the nanozyme activities (GSH oxidation and •OH production) and the photothermal effect with vancomycin, this nanosystem could realize a satisfactory antibacterial outcome against Mu50 (a vancomycin-intermediate *S*. *aureus* reference strain) and *E*. *coli* in vitro. Further, in vivo studies showed that WS_2_QDs-Van@lipo killed bacteria and promoted the healing of the infection area. Additionally, the excellent x-ray attenuation capability of WS_2_QDs made them suitable for computed tomography imaging of infected regions. The strategy of encapsulating drugs in thermosensitive liposomes as shown in this work enables precise drug delivery and can also be used to develop other CDT-involved antibacterial systems.

**Fig. 10.**
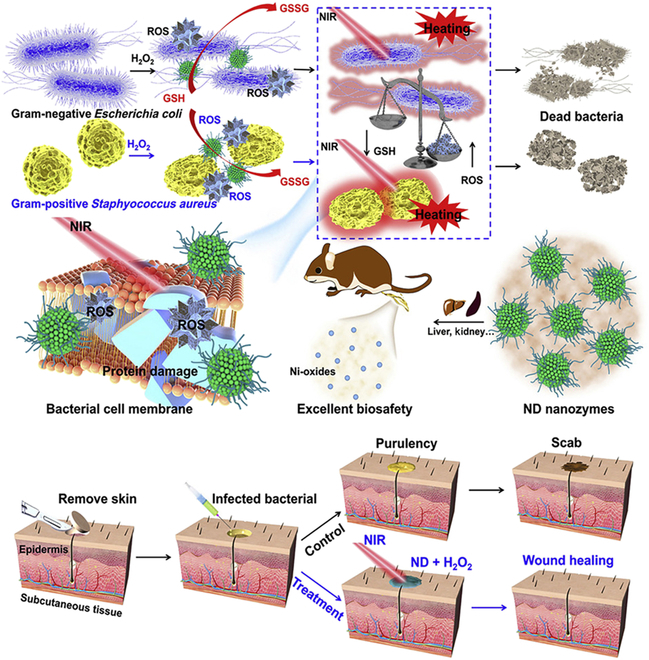
Scheme showing the ND nanozyme with GSH-consuming function for high-efficiency PTT/CDT and its application for in vivo infection treatment. Adapted from [150] under the terms of the Creative Commons CC-BY license.

There are also studies that employ Ni-containing agents for CDT [150,151]. Wang et al. [150] fabricated a monodisperse nickel disulfide (ND) nanozyme via a simple solvothermal method, and the as-prepared ND nanozyme had excellent NIR light absorption, strong photothermal conversion ability, and superb POD-like activity (Fig. 10). Specifically, the obtained ND nanozyme as a horseradish peroxidase-like nanozyme could react with exogenous H_2_O_2_ to yield •OH by the Fenton-like reaction. Furthermore, the catalytic capability of the ND nanozyme could be improved by the mild photothermal performance (PTT) for achieving the photothermally improved catalytic antibacterial therapy. The ND nanozyme could also serve as the glutathione peroxidase mimetic and show an outstanding GSH-consuming function, leading to a better antibacterial effect. In vivo tests showed that in the presence of the NIR light irradiation and H_2_O_2_, effective treatment of infections was successfully achieved through combining CDT and PTT. This multifunctional Ni-based nanozyme can also be utilized as a component to construct other therapeutic platforms via which the synergistic antibacterial effect of multiple therapies can be achieved.

Silver, as a widely used antibacterial agent [159], is also a typical metal element for antibacterial CDT [152,153]. Zhou et al. [152] developed an infection microenvironment-activatable nanocatalytic wound dressing (P-MX/AS@LOx), which consisted of electrospun poly(lactic-*co*-glycolic acid) membrane, MXene/Ag_2_S (MX/AS) bioheterojunction, and lactate oxidase (LOx) for infection-induced chronic cutaneous regeneration. In this system, the poly(lactic-*co*-glycolic acid) membrane gradually degraded to release lactate under the infection microenvironment, and LOx consumed lactate to produce abundant H_2_O_2_. Additionally, the MX/AS bioheterojunction in the membrane not only elicited a mild PTT effect and acted as a photosensitive reagent to generate ROS upon NIR light (PDT effect) but also catalyzed the conversion of the produced H_2_O_2_ into •OH via the Fenton-like reaction mediated by Ag(I)/Ag(0) (CDT effect), which resulted in rapid synergistic sterilization. P-MX/AS@LOx also showed a prominent GSH consumption ability because of its excellent ROS production property under irradiation. Besides, Ag^+^ also displayed continuous antibacterial capability for metal ion therapy. Both in vitro and in vivo results showed that this nanocatalytic dressing possessed ideal bactericidal efficiency and suppressed biofilm growth via synergistic CDT/PDT/metal ion therapy/PTT.

Ru-based CDT has also been studied by many researchers. Zhu et al. [154] employed quaternary ammonium chitosan (QCS) to coat RuO_2_ NSs and then developed QCS-RuO_2_ for effectively treating MDR bacterial infections and biofilms. Specifically, 2D RuO_2_ NSs with satisfactory biocompatibility were constructed by using a poly(ethylene glycol) (PEG) template approach. And an antibacterial drug, [Ru(bpy)_2_(tip)]^2+^ (RBT), was then loaded on QCS-RuO_2_ by tapping accumulation and hydrophobic interaction to afford the final product termed QCS-RuO_2_@RBT NSs (abbreviated as SRT NSs). The SRT NSs with POD-mimetic activity could catalyze H_2_O_2_ to yield highly toxic •OH for CDT. Besides, SRT NSs showed an excellent photothermal conversion ability, which could be used for PTT and improve the catalytic capability of SRT NSs. In the biofilm, extracellular DNA could be cleaved by a high level of the generated •OH, thereby disrupting the rigid biofilm. Subsequently, the antibacterial property of RBT was used to remove planktonic bacteria. The chronic *P. aeruginosa* lung infection model further demonstrated the outstanding antibacterial efficiency of SRT NSs. This work develops a promising material for the application of Ru-based nanozymes in the management of biofilm-caused infections.

### Dual metal elements-mediated CDT

Despite its excellent antibacterial therapeutic effect, the CDT mediated by single metal ions has some limitations, some of which are listed in Table 2. For instance, the activity of Fe^2+^ is remarkably affected by the environmental pH in the Fenton reaction, and the optimum pH range for the Fe-mediated Fenton reaction process is 2 to 4 [15]. Therefore, the different wound environments of bacterial infections might not meet the optimum pH conditions for the Fenton reaction and limit the therapeutic effect of Fe-mediated CDT. Therefore, in some studies, 2 kinds of metal ion redox pairs are introduced in 1 type of material for antibacterial CDT. In this part, we will introduce the strategies in which 2 kinds of metal elements are utilized for CDT via Fenton and/or Fenton-like reactions.

**Table 2.** Advantages and disadvantages of different elements for antibacterial CDT.

Metal element for antibacterial CDT	Advantages	Disadvantages
Fe	Various sources, high catalytic activity, and low cost	Narrow optimal pH range (2–4) for the Fenton reaction
Cu	A broader pH range for the Fenton-like reaction, high catalytic activity, and wound healing promotion capacity	Potential of heavy metal poisoning
Mn	Manganese oxides have the advantages of simple preparation, low cost, and low biological toxicity; in addition, the existence of several valence states of Mn makes manganese oxide an excellent catalyst.	Possible requirement of HCO_3_^−^
Co	High catalytic activity	Toxicity of excessive Co ions
Ni	Good biocompatibility, prominent photothermal performance (for promoting CDT), and excellent biodegradability	Complexes containing Ni^2+^ have short triplet lifetimes and a weak ROS generation ability
Ag	Inherent antibacterial effect	Requirement of increased H^+^ amount in the environment
Ru	High catalytic activity and stability	High cost

**Fig. 11.**
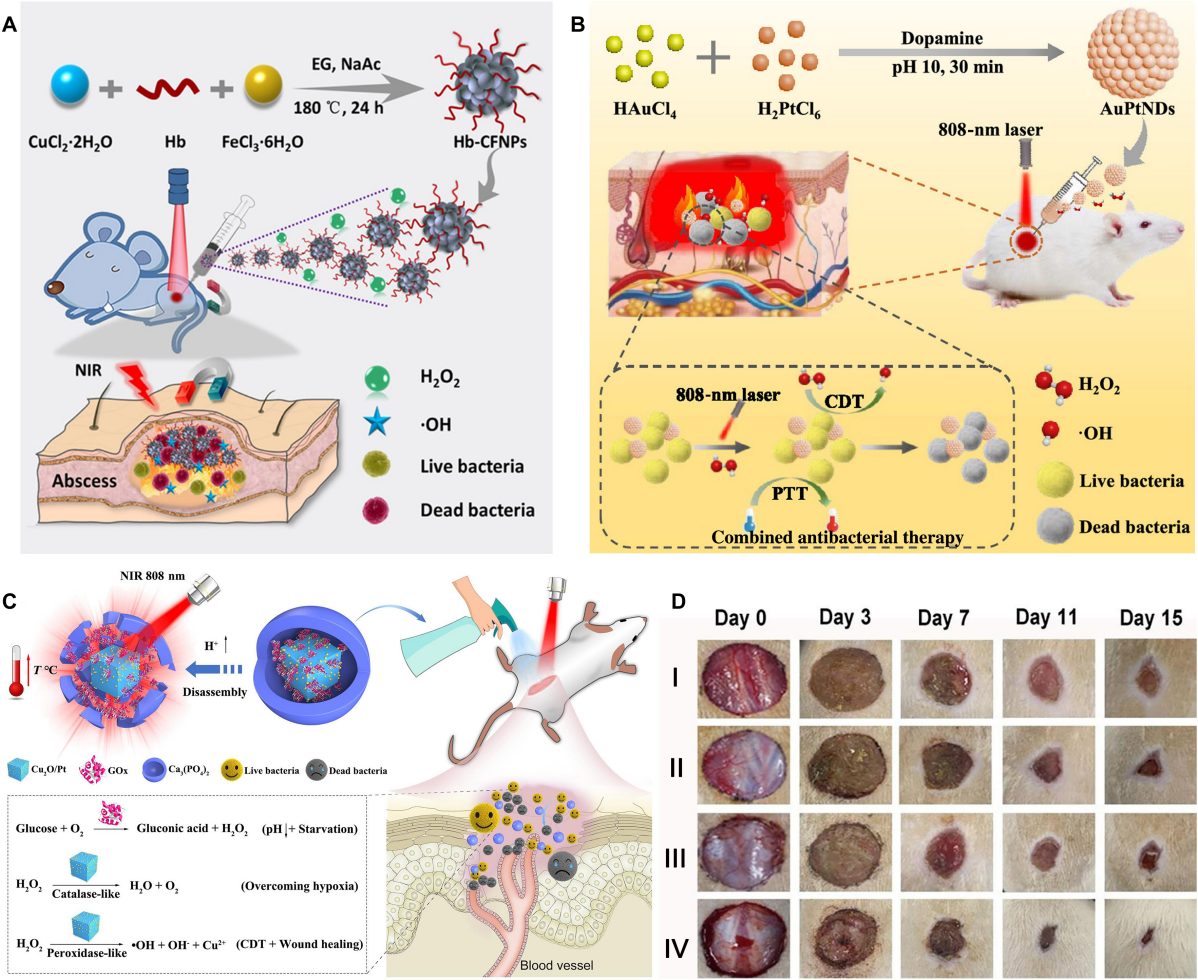
(A) Synthesis of Hb-CFNPs and their antibacterial application via the synergy of CDT and PTT. Adapted from [38] with permission. Copyright 2019, American Chemical Society. (B) Scheme showing the construction of AuPtNDs for combined CDT/PTT. Adapted from [166] with permission. Copyright 2021, American Chemical Society. (C) Schematic representation of the CuPt-GOx-CaP nanoreactor and its use for promoting diabetic wound healing via synergistic PTT, ST, and CDT-mediated sterilization. (D) Photographs of the wounds in diabetic rats subjected to various treatments (I: phosphate-buffered saline [PBS]; II: Cu_2_O/Pt nanozyme; III: CuPt-GOx-CaP; IV: CuPt-GOx-CaP + glucose + NIR). (C and D): Adapted from [167] with permission. Copyright 2022, Elsevier.

Many dual metal elements-mediated CDT strategies are based on copper/iron [38,160–164]. For example, Liu et al. [38] prepared hemoglobin (Hb)-functionalized copper ferrite NPs (termed as Hb-CFNPs) to fabricate a CDT/PTT system for the elimination of bacteria (Fig. 11A). The Fe^2+^/Fe^3+^ and Cu^+^/Cu^2+^ redox pairs in CFNPs catalyzed the convertion of H_2_O_2_ to generate •OH via Fenton and Fenton-like reactions (CDT effect). The •OH could cause the oxidative damage to the bacterial cell membrane, enhancing the sensitivity of the bacteria to heat. Besides, the Cu^2+^ and Fe^3+^ on the Hb-CFNP surface could react with GSH, leading to the GSH consumption. The Hb could improve the dispersion and biocompatibility of CFNPs and enhance the therapeutic efficiency via the Fenton reaction mediated by Hb and H_2_O_2_. Under the NIR light irradiation, Hb-CFNPs with a considerable photothermal conversion ability could generate hyperthermia, which induced the death of the damaged bacteria. Furthermore, because of the excellent magnetic property of Hb-CFNPs, their photothermal efficiency can be improved by about 20 times through magnetic enrichment, and may have beneficial for achieving an ideal bactericidal effect at very low drug doses. Both in vitro and in vivo data proved the outstanding antibacterial effect and biosafety of Hb-CFNPs. Overall, this work realizes enhanced •OH production using 2 Fenton reagents (Hb and CFNPs) and improves the PTT effect by magnetic enrichment, and may have implications for developing synergistic antibacterial platforms in the future. In addition, Guo and coworkers [160] constructed a type of dual transition metal-based nanomaterial (CuFe_5_O_8_ nanocubes [NCs]) for combating biofilms. The CuFe_5_O_8_ NCs could react with H_2_O_2_ in the biofilm and yield massive •OH via the Fenton and Fenton-like reactions. The generated •OH could destroy the extracellular DNA that is important to the stability of biofilm [165], thereby undermining the rigid biofilm. Furthermore, a small amount of •OH could also be generated by CuFe_5_O_8_ NCs outside the biofilm that had a relatively higher pH value and a lower H_2_O_2_ level, which could effectively reverse the immunosuppressive microenvironment via inducing the macrophage polarization to the M1 phenotype. Then, the exposed bacteria and biofilm fragments were persistently eliminated due to the cooperation of •OH and proinflammatory immunity. In vivo experiments showed that the CuFe_5_O_8_ NCs could suppress biofilm formation and promote the elimination of mature biofilms. This work offers an efficacious antibiofilm treatment strategy via the combination of immunomodulation and CDT. Zhang et al. [161] devised a photoactivated copper ferrite (CuFe_2_O_4_) heterojunction that was coated on a polyetheretherketone implant via hydrothermal treatment and π–π stacking. To improve the NIR light absorption and the following phototherapy outcome of CuFe_2_O_4_, the authors introduced 2D graphene oxide (GO) to fabricate the CuFe_2_O_4_/GO heterojunction. The CuFe_2_O_4_/GO-functionalized sulfonated polyetheretherketone with a multimodal bactericidal effect and boosted osteogenicity was termed SP-P-CFN/GO. The cycling of Fe(III)/Fe(II) and Cu(II)/Cu(I) in the CuFe_2_O_4_ heterojunction could continuously catalyze H_2_O_2_ to toxic •OH via Fenton and Fenton-like reactions in a bacterial infection microenvironment and the yielded Cu(II) and Fe(III) could deplete GSH, thus achieving antibacterial cascade reactions. Besides, under the NIR light illumination, the CuFe_2_O_4_/GO heterojunction could realize the PDT process during which the electrons were separated with holes and were captured by O_2_ to produce ^1^O_2_. The SP-P-CFN/GO also possessed an outstanding photothermal effect that was used for NIR light-triggered PTT. In vitro and in vivo results confirmed that the SP-P-CFN/GO-mediated CDT/PDT/PTT led to the satisfactory antibacterial effect. Furthermore, the heterojunction coating could enhance the tissue production and osteogenesis after antibacterial treatment because the release of micronutrient elements (Fe and Cu) was remarkably augmented when the SP-P-CFN/GO implant was under NIR light, causing photo-enhanced osteogenicity. This work develops a system to endow orthopedic implants with the functions of osteogenicity and bacteriostasis to combat recalcitrant implant-associated infections through photo/Fenton strategy.

Additionally, other metal elements have also been utilized to realize 2-metal-ion-based CDT [166–171]. For example, Zhang et al. [166] synthesized ultrasmall gold–platinum nanodots (AuPtNDs) via a one-step approach employing dopamine as a stabilizer and reducing reagent (Fig. 11B). These nanodots displayed satisfactory POD-like activity and a higher affinity for H_2_O_2_ than that of horseradish peroxidase. In the presence of H_2_O_2_, AuPtNDs could effectively catalyze H_2_O_2_ to produce •OH, causing oxidative damage of bacterial cells. The researchers speculated that both Pt and Au played the same important role in the catalytic activity of AuPtNDs. AuPtNDs also had a superb photothermal conversion capability and strong photothermal stability and could transform light energy into heat upon the 808-nm laser irradiation, leading to the death of damaged bacteria without disturbing normal tissues. The in vivo experiments indicated that the nanodots together with externally applied H_2_O_2_ could effectively promote the recovery of the bacterially infected wounds upon 808-nm light irradiation. Besides, in the study of Wang and coworkers [167], the Cu_2_O/Pt nanozyme decorated with GOx was mineralized with calcium phosphate (CaP) to obtain CuPt-GOx-CaP through a one-step biomimetic mineralization approach for healing diabetic wounds (Fig. 11C). CaP is an ideal nanocarrier used in pH-responsive drug delivery because it is stable under neutral pH and can dissolve in the acidic wound microenvironment, leading to the release of the Cu_2_O/Pt nanozyme and GOx. GOx could oxidize glucose, leading to the decrease of the glucose level for ST. The H_2_O_2_ produced in the glucose oxidation process was then converted into •OH, which was caused by the Cu_2_O/Pt nanozyme with the POD activity for CDT. Simultaneously, the Cu_2_O/Pt nanozyme with catalase-mimicking activity could decompose H_2_O_2_ to generate O_2_, which alleviated the hypoxia stress induced by the diabetic wound infection and promoted glucose depletion. The enhanced acidity caused by the GOx-catalyzed production of gluconic acid then in turn promoted the collapse of the CuPt-GOx-CaP nanozyme and the release of copper ions. The released copper ions could contribute to the enhanced angiogenesis. Finally, the Cu_2_O/Pt nanozyme with good photothermal conversion ability could elevate the temperature of the wound microenvironment under 808-nm light irradiation. The generated heat then improved the activity of Cu_2_O/Pt nanozyme and GOx, achieving synergistic PTT/ST/CDT. This multifunctional nanoreactor could realize the treatment against bacteria-infected diseases, which was proved in the *S*. *aureus*-infected diabetic rat models (Fig. 11D). To sum up, this work employs CaP as a pH-responsive nanocarrier, and develops a type of nanomaterial that integrates the depletion of glucose, the generation of bactericidal •OH, and the promotion of wound healing.

## Conclusions and Perspectives

Generally, the potential of antibacterial CDT in future clinical applications can be discussed from 2 aspects. On the one hand, for the infection treatment, the toxic •OH produced during CDT process can cause oxidative stress for bacterial killing. On the other hand, for the wound healing, metal ions introduced in the CDT (such as Cu and Zn ions) have been reported to participate in some biological processes related to wound repair [172,173]. In this review, we have introduced the reported CDT-involved antibacterial strategies mediated by different metal (Fe, Cu, Mn, Co, Mo, Pt, W, Ni, Ag, Ru, V, Au, and Zn)-containing materials such as nanomaterials and hydrogels in detail. These treatments include single CDT and the combined use of CDT and other therapies (phototherapy, ST, chemical therapy, etc.). Many combination strategies have proved that the introduction of other therapies can promote CDT and achieve the “1 + 1 > 2” effects. For example, the elevated temperature caused by PTT can improve the reaction efficiency of CDT, thus achieving satisfactory synergy. In these treatments, the multidrug resistance problem of bacteria can be solved efficiently due to the reduced or even no use of antibiotics. In addition, some materials show excellent antibiofilm ability, indicating their potential in future practical applications.

However, we believe that there is still some room for the future improvement in this field. First, since H_2_O_2_ is one of the important substrates of Fenton/Fenton-like reactions, the therapeutic effect of antibacterial CDT will be limited by the insufficient H_2_O_2_ amount. Many strategies mentioned in this review solved this problem through adding exogenous H_2_O_2_ (that is, H_2_O_2_ and CDT drugs were administered together). However, this is not a convenient method. How to realize efficient and safe self-production of H_2_O_2_ in the infection regions should be considered in the future. Second, although several metal elements like Co, Mo, Pt, W, Ni, Ag, Ru, and Zn have been employed in designing diverse antibacterial CDT reagents, these elements have not been fully researched and widely adopted in practical antibacterial treatments. Meanwhile, the CDT strategies mediated by dual metals are relatively rare. Therefore, more materials based on other single metal elements and dual metal elements with satisfactory Fenton/Fention-like reaction activities need to be explored. Third, for realizing more accurate antibacterial treatments with fewer side effects, it is necessary to design intelligent materials with the capabilities of on-demand drug release as well as bacterial theranostics. Different stimulation approaches (e.g., US, light, and magnetic field) can be employed for selectively releasing CDT reagents in the infection regions. Fourth, future researchers can pay more attention to the wound healing process and combine CDT agents with materials that promote the repair of damaged tissues (e.g., growth factors [174]). Fifth, since the traditional CDT process needs to be achieved through introducing metal ions that may result in safety problems, some CDT strategies mediated by nonmetal elements have been designed for tumor treatment. Xu et al. [175] developed a biomimetic nanozyme (termed NC@GOx NPs) through decorating GOx on nitrogen-doped carbon NPs via electrostatic interaction for ST-enhanced CDT/PTT. However, the research on the development of metal-free antibacterial CDT agents is still rare. The design of metal-free materials with lower cytotoxicity can open a new door for realizing safe CDT-based antibacterial treatments. Sixth, the types of bacteria selected in the above researches are not rich enough. Although some researchers have developed antibacterial materials for some MDR bacteria, more materials have only been proved to possess satisfactory killing efficacy toward *S*. *aureus* and/or *E*. *coli*. Consequently, bioactive materials capable of treating other types of bacteria should be developed in the future. Seventh, considering the facts that CDT has been widely used in anticancer treatments and the surgical removal of tumor has the risk of postoperative infection, we think it is necessary to develop CDT agents suitable for both anticancer and antibacterial applications, and it is also important to construct suitable CDT agent-containing dressings /filling agents for facilitating postoperative wound healing. Finally, for the clinical application of CDT, more biological safety evaluations of the designed CDT materials should be conducted, especially on the long-term safety, metal toxicity, and in vivo degradability.

Collectively, to date, many CDT agents have exhibited great application potential in the antibacterial field. Nevertheless, more research is still needed for the development of novel metal-based and metal-free materials, the enhancement of the CDT effect, and the safety improvement of the antibacterial agents. We believe that more CDT materials and strategies can be employed for desirable anti-infection treatments in the near future.
